# Evaluating species distribution model predictions through time against paleozoological records

**DOI:** 10.1002/ece3.70288

**Published:** 2024-10-22

**Authors:** Ignacio A. Lazagabaster, Chris D. Thomas, Juliet V. Spedding, Salima Ikram, Irene Solano‐Regadera, Steven Snape, Jakob Bro‐Jørgensen

**Affiliations:** ^1^ Department of Evolution, Ecology and Behaviour University of Liverpool Liverpool UK; ^2^ Centro Nacional de Investigación Sobre Evolución Humana CENIEH Burgos Spain; ^3^ Leverhulme Centre for Anthropocene Biodiversity, Department of Biology University of York York UK; ^4^ Department of Archaeology, Classics and Egyptology University of Liverpool Liverpool UK; ^5^ Department of Sociology, Egyptology and Anthropology The American University in Cairo New Cairo Egypt; ^6^ Max Planck Institute for Meteorology Hamburg Germany

**Keywords:** accuracy, hartebeest, Holocene, model evaluation, past, SDM, sensitivity, true skill statistic

## Abstract

Species distribution models (SDMs) are widely used to project how species distributions may vary over time, particularly in response climate change. Although the fit of such models to current distributions is regularly enumerated, SDMs are rarely tested across longer time spans to gauge their actual performance under environmental change. Here, we utilise paleozoological presence/absence records to independently assess the predictive accuracy of SDMs through time. To illustrate the approach, we focused on modelling the Holocene distribution of the hartebeest, *Alcelaphus buselaphus*, a widespread savannah‐adapted African antelope. We applied various modelling algorithms to three occurrence datasets, including a point dataset from online repositories and two range maps representing current and ‘natural’ (i.e. hypothetical assuming no human impact) distributions. We compared conventional model evaluation metrics which assess fit to current distributions (i.e. True Skill Statistic, TSS_c_, and Area Under the Curve, AUC_c_) to analogous ‘paleometrics’ for past distributions (i.e. TSS_p_, AUC_p_, and in addition Boyce_p_, F2‐score_p_ and Sorensen_p_). Our findings reveal only a weak correlation between the ranking of conventional metrics and paleometrics, suggesting that the models most effectively capturing present‐day distributions may not be the most reliable to hindcast historical distributions, and that the choice of input data and modelling algorithm both significantly influences environmental suitability predictions and SDM performance. We thus advocate assessment of model performance using paleometrics, particularly those capturing the correct prediction of presences, such as F2‐score_p_ or Sorensen_p_, due to the potential unreliability of absence data in paleozoological records. By integrating archaeological and paleontological records into the assessment of alternative models' ability to project shifts in species distributions over time, we are likely to enhance our understanding of environmental constraints on species distributions.

## Graphical abstract

Species distribution models (SDMs) are commonly used to project species distribution changes on the face of climate change, but their long‐term performance is rarely tested. To address this, the authors employed paleozoological records to independently assess SDM accuracy. They focused on modelling the historical distribution of the hartebeest and compared the models' ability to predict past presences and absences in the archaeological record. The results demonstrated a lack of strong correlation between present‐day and historical model performance, emphasising the influence of input data and modelling choices on SDM accuracy and highlighting the value of using paleontological and archaeological records for model assessment.

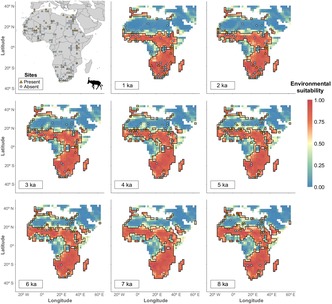

## 1 INTRODUCTION

Species distribution models (SDMs) have emerged as a valuable and versatile tool that is now widely applied in multiple research domains (Araújo et al., 2019; Austin, 2007; Elith & Leathwick, 2009; Franklin et al., 2013; Payne & Bro‐Jørgensen, 2016a; Smith et al., 2013; Svenning et al., 2011; Varela et al., 2011). These models predict the suitable geographic area for a species based on the environmental conditions (e.g., climate, vegetation, topography, soil) of the localities where the species is known to occur. In historical biogeography, SDMs have proven highly useful for understanding the impact of past climatic and environmental changes on the distribution and diversification of fauna and flora (Lazagabaster et al., 2021; Lentini et al., 2018; Martin et al., 2023; Svenning et al., 2011). Furthermore, understanding species' distribution shifts in response to climate change may assist in developing optimal conservation and management plans, especially for species vulnerable to extinction and invasive taxa (Barnosky et al., 2017; Dietl et al., 2015; Jeschke & Strayer, 2008; Lima‐Ribeiro et al., 2017; Payne & Bro‐Jørgensen, 2016b; Svenning et al., 2011).

Despite their wide use, the reliability of SDM outputs depend heavily on the quality of occurrence data (Araújo et al., 2019; Barbet‐Massin et al., 2012; Duputié et al., 2014; Fourcade, 2016; Lobo et al., 2010; Varela et al., 2011). Ideally, the distributional data should come from comprehensive sampling (Tessarolo et al., 2014) so that it captures the environmental conditions of all the habitats that the species occupies (Barbet‐Massin et al., 2010; Varela et al., 2009). Online data repositories are the main sources of occurrence records but they often contain heterogeneous and biased datasets (Fourcade, 2016), for example, due to bias in human observations towards certain locations, such as national parks and sites of scientific studies, and differences in museum data digitalisation and sharing data policies (Beck et al., 2014).

Another critical aspect of SDM analysis is the choice of modelling algorithms, as these determine the relationship between the presence–absence dataset and the chosen environmental variables, thus forming the basis for generating habitat suitability predictions (Elith & Graham, 2009; Hallgren et al., 2019; Li & Wang, 2013; Pearce & Ferrier, 2000; Tsoar et al., 2007). Various algorithms are available, each with its advantages and disadvantages (Carlson, 2020; Elith & Leathwick, 2009; Hallgren et al., 2019; Li & Wang, 2013; Pearce & Ferrier, 2000), and it is critical to understand the potential and pitfalls of the various modelling procedures.

To assess the appropriateness of using alternative occurrence datasets and model algorithms, it is essential to evaluate the performance of SDMs in terms of their reliability and applicability across different contexts. Multiple evaluation metrics comparing the predicted distributions with the actual species occurrences have been developed (Allouche et al., 2006; Fourcade et al., 2018; Wunderlich et al., 2019). These evaluation metrics are categorised into threshold‐dependent and threshold‐independent measures.

Threshold‐dependent metrics require the conversion of continuous probability outputs of SDMs into binary presence‐absence predictions based on a threshold value. Among threshold‐dependent metrics, the True Skill Statistic (TSS; Allouche et al., 2006) is one of the most common in the literature. TSS balances the True Positive Rate (TPR), also known as Sensitivity or Recall, and the True Negative Rate (TNR), also known as Specificity. TPR measures the proportion of actual positives that are correctly identified as such by the model, thus focusing on the model's effectiveness in detecting positive outcomes. By contrast, TNR measures the proportion of actual negatives that are correctly identified as such by the model, thus focusing on the model's ability to detect negative outcomes. TSS is particularly valued for its independence of prevalence, which makes it robust in scenarios where the species' presence is rare or unevenly distributed (Allouche et al., 2006; but see Wunderlich et al., 2019). Additionally, Accuracy and Precision are often reported alongside TSS to give a comprehensive view of model performance. Accuracy represents the overall proportion of true results (both true positives and true negatives) among the total predictions made, providing a general measure of how well the model performs across all predictions. Meanwhile, Precision measures the proportion of detected presences that are true, highlighting the reliability of positive predictions made by the model (Liu et al., 2011).

Other notable threshold‐dependent metrics include F‐scores and the Sørensen similarity index. The F‐scores combine TPR and Precision, providing a balanced measure of model fitness by considering both false positives and false negatives (Liu et al., 2011). This metric is especially useful in situations where failing to detect a presence (false negative) is as costly as incorrectly signalling a presence (false positive). The Sørensen index is a derivative of the Jaccard index, which is calculated by taking the number of sites where both the model and observations agree on the presence of the species and dividing this by the total number of sites where the species is predicted or observed to be present (Pottier et al., 2013). By focusing solely on the presence records, the Sørensen Index is particularly useful when knowing where a species is likely to be found is more critical than knowing where it is not.

Among threshold‐independent metrics, the Area Under the Curve (AUC) of the Receiver Operating Characteristic (ROC) curve is the most used. AUC assesses the model's capability to distinguish between presence and absence locations over all potential threshold values. Despite its widespread use, AUC may be influenced by the spatial distribution of presence and absence data points (Lobo et al., 2008). Another significant threshold‐independent metric is the Boyce Index, which evaluates how effectively the model predicts presences as compared to random expectation. This metric is particularly valuable for analysing presence‐only data (Jiménez & Soberón, 2020). While AUC focuses on the model's ability to differentiate presences from pseudoabsences, the Boyce Index highlights the model's capacity to identify presences in areas of higher habitat suitability (Radomski et al., 2022).

Evaluation metrics are typically used to assess the ability of SDMs to predict areas that are known to be suitable at present. However, SDMs are rarely tested across sufficient time spans and degrees of environmental change (Inman et al., 2018; Myers et al., 2015) to properly understand their hindcasting performance. When models are applied to past or future conditions—where environmental variables may deviate significantly from present‐day norms—their effectiveness can vary (Inman et al., 2018; Varela et al., 2009; Werkowska et al., 2017). SDMs typically use input data from distributions reflecting the species realised niche at present, but the realised niche is influenced by the dynamic interplay of environmental conditions, biotic interactions, such as competition and predation, and dispersal constraints (Cranston et al., 2024). These factors collectively shape the realised niche within the broader fundamental niche and are inherently dynamic, potentially changing over time (Soberón, 2010). Establishing these responses is particularly challenging when projecting to past or future climates that have no present‐day analogue (Varela et al., 2009). The assumption of niche conservatism—that a species' ecological niche remains stable over time—can further be challenged by adaptive change over evolutionary time (Losos, 2008; Pearman et al., 2014; Stigall et al., 2014; Stigall, 2012). Validating SDM predictions with truly independent data, such as fossil records, offers a solution to interpret the realism of the scenarios generated (Lazagabaster et al., 2021; Lentini et al., 2018; Lima‐Ribeiro et al., 2017; Martin et al., 2023; Roberts & Hamann, 2012; Smith et al., 2013).

The main goal of this study is to evaluate the performance of alternative combinations of presence/absence data and modelling algorithms by using measures of model efficiency that compare predicted geographic distributions in the past against actual changes in species distributions over relatively long periods of time as estimated from archaeological evidence. To illustrate our approach, we investigated the historical biogeography of the hartebeest *Alcelaphus buselaphus*, a large widespread savanna‐adapted African antelope. We calculated various evaluation metrics that compare model‐predicted probabilities of occurrence with the hartebeest's modern distribution and with its distribution over the past 8000 years, as deduced from the paleozoological record from Holocene Africa. The combinations of input data and modelling algorithms that represent the hartebeest's paleozoological record most effectively are assumed to provide the most realistic historical reconstructions of suitable environments that can be generated with the available data. This allowed us to test whether the models that were best at predicting the hartebeest's modern distribution also outperformed other models in predicting its historical distribution.

## 2 MATERIALS AND METHODS

### 2.1 Test case scenario: the historical biogeography of the hartebeest

The hartebeest (143–177 kg, depending on subspecies) is an African antelope that lives in diverse environments in terms of primary production, habitat structure and seasonality; its habitats include sub‐grasslands, bushlands, and woodland savannas (Gosling & Capellini, 2013; Kingdon, 2015). Today, it is confined to sub‐Saharan Africa, but its historical distribution includes large parts of Mediterranean North Africa, the Levant, and the Saharo‐Sahelian region. Dates of extinction from most of the Sahara occur during the mid‐Holocene drying trend following the African Humid Period (AHP, ~11,500–5000 ka), but the now extinct subspecies known as the bubal (*Alcelaphus buselaphus buselaphus*) continued to survive in Mediterranean areas from Morocco to Egypt until the early 20th century when it was extirpated by over‐hunting (Loggers et al., 1992). Like many other large African mammals, hartebeests survive today predominantly in and around protected areas (Gosling & Capellini, 2013; Kingdon, 2015).

### 2.2 Workflow for the species distribution modelling

**FIGURE 1:**
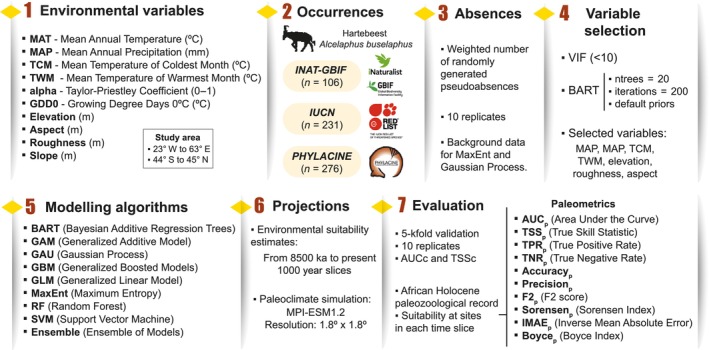
Main components of the analyses followed to model the Holocene geographic distribution of the hartebeest in this study.

We divided the modelling workflow into seven steps focused on: (1) the study area and environmental data, (2) occurrence data, (3) absence data, (4) variable selection, (5) modelling algorithms, (6) hindcasting projections, and (7) model evaluation (Figure 1).

#### 2.2.1 Study area

The study area was selected to encompass the known paleozoological record of the hartebeest, including Africa and the Levant (23° W to 63° E longitude and 44° S to 45° N latitude).

#### 2.2.2 Environmental data

Earth System Models (ESMs) often face challenges in simulating past climatic conditions, which is indeed the case for precipitation patterns in North Africa, where their outputs sometimes diverge from proxy‐derived estimates, such as pollen‐based reconstructions (Braconnot et al., 2012). In this study, we use climate data for the past 7850 years obtained from a transient simulation conducted with the comprehensive ESM MPI‐ESM1.2, with a raster cell size of 1.8° × 1.8° (~200 km^2^; Dallmeyer et al., 2020). Notably, this simulation demonstrates a stronger concordance with precipitation reconstructions derived from pollen records during the African Humid Period (AHP) when compared to simulations within the Paleoclimate Model Intercomparison Project (PMIP) framework.

The variables used include running means over 100 years of monthly mean precipitation (kg/m^2^s) and mean 2 m air‐temperature (°K), both derived from the MPI‐ESM1.2 BIOME 1 model (Dallmeyer et al., 2020; Prentice et al., 1992). We also included the mean temperature of the coldest month (TCM, in°C), the mean temperature of the warmest month (TWM, in°C), the Taylor‐Priestley coefficient (‘alpha’), and the Growing Degree Days based on 0°C (GDD0). The Taylor‐Priestley coefficient is an indicator of moisture availability ranging from 0 to 1 and is calculated as the ratio of actual evaporation to potential evaporation, where values under 0.18 are associated with deserts. The GDD is calculated as the difference between the mean daily air temperature (°C) and a threshold base temperature (0°C in this case).

We used the ice sheet extent data from the CHELSA‐Trace21k paleo‐orographic simulations (Karger et al., 2023) to mask the bioclimatic rasters and to calculate four topographic variables, i.e. elevation, aspect, roughness, and slope. The CHELSA‐Trace21k dataset provides 30 arcsec‐resolution paleo‐orographic reconstructions in 100‐year time steps for the last 21,000 years, created by combining high‐resolution information on glacial cover interpolated with a dynamic ice sheet model (ICE6G) (Karger et al., 2023).

All the topographic and bioclimatic layers were averaged into eight rasters. The raster 600 represents the years 200 to 1000 BP, the raster 1500 represents the years 1001 to 2000 BP, the raster 2500 represents the years 2001 to 3000 BP, the raster 3500 represents the years 3001 to 4000 BP, the raster 4500 represents the years 4001 to 5000 BP, the raster 5500 represents the years 5001 to 6000 BP, the raster 6500 represents the years 6001 to 7000 BP, and the raster 7500 represents the years 7001 to 7850 BP.

#### 2.2.3 Occurrence data

**FIGURE 2:**
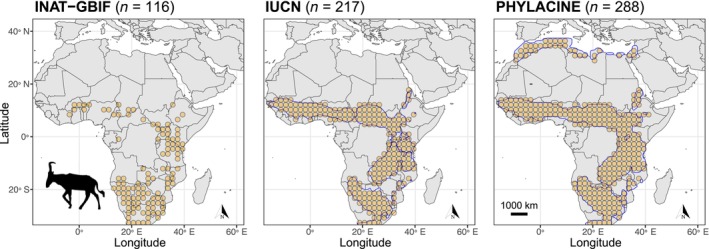
Map of the study area showing the three hartebeest's occurrence datasets used in the construction of species distribution models.

Data relating to the present‐day distribution of hartebeest were obtained from the combination of two open‐access online repositories of sightings and other records, i.e. the iNaturalist (INAT) and the Global Biodiversity Information Facility (GBIF). Visual examination of the INAT‐GBIF occurrences revealed that many observations were heavily clustered (Figure 2 and Figure S1). To reduce biases due to data point clustering, we applied spatial thinning by adjusting the presence data to align with the climate raster's resolution (Velazco et al., 2022). Moreover, we also generated pseudo‐presence data from distribution ranges produced by the International Union of Conservation of Nature (IUCN) Red List of Threatened Species (actual distribution assessed by IUCN experts) and the reconstructed natural ranges provided in the Atlas of Mammal Macroecology v.1.2.1 PHYLACINE dataset, which represent hypothetical distributions in the absence of present and historical human impacts (Faurby et al., 2018).

##### INAT‐GBIF

This dataset was obtained from two online repositories of species observations. First, we downloaded research‐quality‐only occurrences (georeferenced observations verified by two or more experts and backed by photographs) with a coordinate uncertainty <10,000 m from the citizen science website iNaturalist at https://www.inaturalist.org/. The iNaturalist query with taxon name = ‘Alcelaphus buselaphus’ produced 1661 results. We used spatial thinning to reduce the sampling bias inherent to this type of observational datasets; specifically, we adjusted the presence data to the climate raster resolution allowing only one presence point per raster cell, resulting in a total of 92 occurrences. In addition, we obtained occurrence data also from the Global Biodiversity Information Facility (GBIF) at https://www.gbif.org/. The query was filtered to select ‘human observations’ and ‘fossil specimens’ from the year 1800 to the present and with a coordinate uncertainty <10,000 m. The data was cleaned to eliminate coordinate uncertainty, taxonomic problems, and data incongruency. We further selected only data points that were present within the hartebeest's current natural range (from the PHYLACINE dataset, see below) plus an extra 100‐km buffer around this range. Of 812 initial results, the filtered and cleaned dataset included 734 records. After adjusting the presence data to the climate raster resolution, the GBIF dataset had 82 occurrences. We then combined the INAT and GBIF cleaned datasets, and after removing duplicates, the final dataset included 106 occurrences (Figure 2).

##### IUCN

This dataset represents the actual current distribution of the hartebeest as assessed by International Union of Conservation of Nature (IUCN) Red List of Threatened Species experts. Polygon shapefiles are available for download at https://www.iucnredlist.org/. All cells available at the raster resolution within the IUCN polygon were taken as occurrence (=pseudopresence) records (*n* = 231, Figure 2).

##### PHYLACINE

This dataset represents the hypothetical present‐day natural range for the hartebeest as provided by the Atlas of Mammal Macroecology v.1.2.1 PHYLACINE database (Faurby et al., 2018). These natural ranges estimate the current potential distribution of species in the absence of anthropogenic impacts such as extinctions or extirpations. Notably, the authors suggest that, without human influence, hartebeests would likely inhabit most of the northern African Mediterranean coast today. The PHYLACINE estimations are derived from methods that include fossil co‐occurrence modelling and relatively recent historical sightings, so it is anticipated that these ranges would more accurately predict the fossil occurrences of the hartebeest than the IUCN or the INAT‐GBIF, which only include present‐day observations and ranges. The shapefile polygons used were downloaded from https://megapast2future.github.io/PHYLACINE_1.2/. We included all cells within the PHYLACINE polygon at the dataset's raster resolution as occurrence records (*n* = 276, Figure 2).

#### 2.2.4 Absence data

For each of three occurrence datasets, we generated 10 sets of randomly selected (pseudo)absence points (i.e. considering only raster cells with no occurrence points) and one set of background points (i.e. considering all raster cells within the entire study area, including cells with occurrence points). For the INAT‐GBIF dataset, the pseudoabsence points were selected from cells outside 100‐km buffers around each presence point. For the IUCN and PHYLACINE datasets, the absence points were taken outside of the respective polygons. The number of absences equalled the number of occurrences, following the recommendation of weighted sample sizes (Barbet‐Massin et al., 2012). Background data are recommended for presence‐only species distribution modelling algorithms such as MaxEnt (Phillips et al., 2009) and are sometimes used also for other methods, including tree classification and machine learning techniques (Valavi et al., 2022). We selected all raster cells in the study area as background points (*n* = 978, Figure S2).

#### 2.2.5 Variable importance and selection

For each of the three occurrence datasets, we first removed variables with Variance Inflation Factor (VIF) >10 to avoid collinearity between covariates from affecting the results. We removed additional variables by applying the automated stepwise variable set reduction procedure in the Bayesian additive regression trees algorithm (BART), available in the *embarcadero* R library (Carlson, 2020). This procedure eliminates the least informative variable based on the root mean square error (RMSE) for a specified number of model iterations (*n* = 200) and a set number of trees (*n* = 20; based on recommendation of Carlson, 2020, cfr. also Chipman et al., 2010). Normally, there is a strong agreement between the selected variables and those that ranked higher in variable importance (Carlson, 2020), and this was the case also in our analyses. Variable importance was calculated by counting the number of times a given variable is used by a tree split across the full posterior draw of trees after 200 iterations. This method weighs the relative importance of each variable across all decision trees and thus spreads variable contribution across all covariates.

The selected variables, in order of importance, were MAP, TCM, TWM, elevation, and roughness (Figure S3). The relationship between predictor variables and environmental suitability is shown by the partial dependence plots in Figures S4–S6. Partial dependence plots show the response curves of an individual variable in relation to environmental suitability estimations. In all cases, MAP follows approximately a normal distribution, with a peak of environmental suitability values between ~500 and ~1200 mm. The results also indicate that hartebeests prefer habitats with TCM above 10°C and TWM below 28°C. The primary importance of precipitation agrees well with precipitation being the major determinant of vegetation production in tropical areas (Chamaillé‐Jammes & Fritz, 2009).

#### 2.2.6 Model implementation

We modelled potential geographic distribution of the hartebeest using eight modelling algorithms (Figure 1), i.e. BART, generalised linear model (GLM), generalised additive model (GAM), Gaussian process (GAU), support vector machine (SVM), maximum entropy (MaxEnt), random forest (RF), and generalised boosted regression (GBM). The main characteristics of each algorithm are described in Supporting Information S6. BART is implemented in the R library *embarcadero* (Carlson, 2020), with all other SDM modelling algorithms using the *flexsdm* library in R (Velazco et al., 2022). In addition, we constructed an ensemble model by combining all the models except BART (which is not implemented in the *flexsdm* library), using a TSS‐based weighted average approach. The nine resulting modelling frameworks were applied to each combination of the three occurrence datasets with the 10 random pseudoabsence replicates.

To generate simple, easy‐to‐interpret models, GLM was set to work with polynomials of second order only and with no interactions, and correspondingly, the k value (number of knots) in GAM was set to two; this is based on the consideration that whereas n‐ or U‐shaped quadratic relationships between explanatory variables and probability of occurrence often make biological sense, this is not the case for higher order polynomials. For both MaxEnt and GAU, the models were fitted with presences and background points but were validated against presences and pseudoabsences (see model evaluation section below). This procedure makes MaxEnt and Gaussian processes comparable to other presence‐absence models (Velazco et al., 2022). The rest of the algorithms, which usually produce more complex models, were run with the default R flexsdm settings. Finally, BART models were run with the default parameters in the library e*mbarcadero* (Carlson, 2020), i.e. 200 trees and 1000 back‐fitting Markov chain Monte Carlo (MCMC) iterations and discarding 20% as burn‐ins.

All combinations of occurrence‐pseudoabsence dataset and modelling algorithm were used to project distributions for eight past time slices, totalling 2160 projections. The projections were then evaluated against the paleozoological record to compare their performance (Sections 2.3 and 2.4).

### 2.3 The paleozoological dataset

We obtained paleozoological presence/absence data for the Holocene from the compilation of dated faunal remains from Africa in Phelps et al. (2020) (https://doi.pangaea.de/10.1594/PANGAEA.904942). Note that this dataset is limited to the African continent and does not include areas of the Middle East (including Palestine and Israel), where the hartebeest is known to have been present in the Holocene (Tsahar et al., 2009). The dataset includes earliest and latest possible dates derived from calibrated radiocarbon dates or known cultural or typological periods, and we included each occurrence record in all of the time slices with which the reported date interval overlapped either fully or partially. For this assignment process, we developed an R function *dates_to_time_slices()*, which allows the user to either select the full time interval, as in our analysis (option = ‘interval’), or just the midpoint of each interval (option = ‘midpoint’) when assigning records to time slices.

In our analysis, the relatively low resolution of the environmental raster meant that certain sites, particularly coastal locations in eastern and southern Africa, were inadvertently omitted due to their placement outside of the information‐containing pixels. To prevent such data loss and ensure comprehensive coverage, we also developed a custom R function *coordinates_to_nearest_valid()*, which systematically adjusts the coordinates of the occurrence data by aligning them with the nearest valid raster cells, thereby preserving as much data as possible.

### 2.4 Model evaluation

#### 2.4.1 Fit to present‐day distribution

Model performance was evaluated with AUC_c_ and TSS_c_ using a 5k‐fold cross‐validation approach with 10 replicates (Allouche et al., 2006; Márcia Barbosa et al., 2013). TSS was rescaled to the 0–1 range to facilitate comparison with other evaluation metrics (Barbosa, 2015). AUC and TSS values <0.4 are considered poor, 0.4–0.8 useful, and >0.8 excellent (Zhang et al., 2015). Independent thresholds were calculated for each model such that the sensitivity and specificity were maximised (Figures S7 and S8). These thresholds represent cut‐off values with which continuous environmental suitability projections are converted to binary scores with discrete favourable or unfavourable areas.

#### 2.4.2 Fit to past distribution

We calculated both threshold‐dependent and threshold‐independent metrics to evaluate models fit to past distribution (Table 1). The calculation of threshold‐dependent metrics is based on the construction of a confusion matrix (Table 2), where *a* is the number of fossil localities for which presence was correctly predicted by the model (true positives); *b* is the number of localities in which remains of the species were not found but the model predicted suitable habitats (false positives); *c* is the number of localities for which the species was found but the model predicted unsuitable habitats (false negatives); and *d* is the number of localities for which absence was correctly predicted by the model (true negatives) (Allouche et al., 2006). From this confusion matrix, we calculated the following metrics: True Positive Rate (TPR_p_), True Negative Rate (TNR_p_), False Positive Rate (FPR_p_), False Negative Rate (FNR_p_), True Skill Statistic (TSS_p_), Accuracy_p_, Precision_p_, F2 Score_p_, and Sorensen_p_.

**TABLE 1:** Description of evaluation metrics included in the *paleo.eval* function and used in this study.

Metric	Formula	Description
Threshold‐dependent		
True positive rate (TPR)	$\frac{a}{a+c}$	Also known as Sensitivity or Recall, measures the proportion of actual positives (presence of the taxon) that are correctly identified by the model
True negative rate (TNR)	$\frac{d}{b+d}$	Also known as Specificity, measures the proportion of actual negatives (absence of the taxon) that are correctly identified
False positive rate (FPR)	$\frac{b}{b+d}$	Measures the proportion of actual negatives that are incorrectly classified as positives
False negative rate (FNR)	$\frac{c}{a+c}$	Measures the proportion of actual positives that are incorrectly classified as negatives
True skill statistic (TSS)	TPR+TNR−1	Quantifies the model's ability to avoid both false positives and false negatives
Accuracy	$\frac{a+d}{n}$	Quantifies the overall ability of the model to correctly classify both presences and absences
Precision	$\frac{a}{a+b}$	Indicates the proportion of positive identifications that were actually correct
Prevalence	$\frac{a+c}{a+b+c+d}$	Reflects the proportion of actual positives in the dataset
F2 score	$2*\frac{\text{precision}*\mathrm{TPR}}{\text{precision}+\mathrm{TPR}}$	Is the harmonic mean of precision and recall, providing a balance between them
False discovery rate (FDR)	$\frac{b}{a+b}$	Measures the proportion of positive results that were false positives
Sorensen	$2*\frac{a}{c+\left(2*a\right)+d}$	Similar to F1 Score, measures the similarity between predicted and observed presences
Jaccard	$\frac{a}{a+c+d}$	Another similarity measure between the predicted and observed data
Odds ratio (OR)	$\frac{a*d}{b*c}$	Assesses the odds of prediction success relative to prediction failure
Kappa	$\frac{P_{o}-P_{e}}{1-P_{e}}$	Where *P*_o_ is the observed agreement, and *P*_e_ is the expected agreement under independence. Measures inter‐rater agreement for qualitative items, adjusting for chance agreement
Threshold‐independent		
Area under the curve (AUC)	$\frac{R-n_{1}*\left(n_{1}+1\right)/2}{n_{1}*n_{2}}$	From the Receiver Operating Characteristic (ROC) analysis, where *R* is the sum of ranks of positives, *n*_1_ is the number of positives, and *n*_2_ is the number of negatives. It measures the model's discriminative ability, i.e., its capability to correctly classify observations into presence or absence categories across all possible threshold values
Boyce	$B=\rho \left(S,E\right)$	Simplified conceptual formula where ρ is Spearman's rank correlation coefficient, S are the mid‐points of suitability bins, and E is the cumulative sum of observed presences up to each bin. It evaluates the model's predictive performance by comparing the observed presences against a background of predictions
Index of model absolute error (IMAE)	$\frac{\sum i=\text{real}-\text{pred}}{n}$	Where, ∣real_*i*_ − pred_*i*_∣ is the absolute error for each data point and is the total number of observations in the dataset. It quantifies the absolute deviation of predictions from reality, offering a normalised error rate across the dataset

**TABLE 2:** Confusion matrix used to calculate the thresholded paleometrics. After Allouche et al. (2006), but using paleozoological records as validation sites.

	Paleozoological record	
	Presence	Absence
SDM projections		
Presence	a (correctly classified presence)	b (incorrectly classified absence)
Absence	c (incorrectly classified presence)	d (correctly classified absence)

The threshold independent measures included the Area Under the Curve (AUC_p_) and the Boyce Index (Boyce_p_). AUC_p_ was calculated through the *roc* and *auc* functions in the R library ‘pROC’ (Robin et al., 2011), whereas Boyce_p_ was obtained using the *evalContBoyce* function in the enmSdmX package (Hirzel et al., 2006; Smith, 2023). We also computed the Index of Model Absolute Error (IMAE_p_) to quantify the absolute deviation of model predictions from actual observed outcomes. This metric calculates the absolute difference between the predicted probability and the actual observed value for each data point, sums these absolute errors and divides the sum by the total number of observations to achieve a normalised measure.

To facilitate the calculation of these metrics, which we refer to as ‘paleometrics’, we provide the R script with the function *paleo.eval* (Appendix S1, Table S1). This function requires a raster of model predictions for the time slices to be evaluated, the specification of a threshold value, and a dataset indicating latitude and longitude of paleozoological localities, species composition, and time range. We computed paleometrics using the *paleo.eval* function to compare environmental suitability projections obtained using different occurrence datasets and modelling algorithms for each of the eight time slices to assess how datasets and algorithms influenced the reliability of our results (Figures S10–S18).

All metrics were scaled to range from 0 to 1 to allow standardised comparisons across different models. The statistical comparisons were done utilising non‐parametric Kruskal–Wallis and post‐hoc Dunn tests with Bonferroni correction to ensure robust comparisons. All analyses were performed in R v.4.3.1. and RStudio v.2023.06.2‐561.

#### 2.4.3 Sensitivity analysis

Not all organisms present in an ecosystem are preserved, and not all preserved remains are discovered (Behrensmeyer et al., 2000; Jablonski et al., 1986), therefore paleozoological records are inherently biased taxonomically, spatially, and temporally (Behrensmeyer et al., 2000; Inman et al., 2018; Lloyd et al., 2012). Because absence of a taxon at a specific site and time in the paleozoological record does not necessarily indicate its true absence from the area, false positives (parameter *b* in the confusion matrix) can skew results towards artificially low values of the TNRp and thereby reduce estimates of paleometrics that incorporate evaluation of absences, such as TSSp and AUCp. In the paleozoological dataset used in this study, there is a disproportionate number of localities with only few species (Figure S19) and we observed that hartebeest presence was recorded more times at sites were four species or more had been documented. Thus, we conducted a sensitivity analysis to examine the potential effect of sampling bias in the paleozoological dataset. Sites with the lowest recorded species richness were progressively eliminated and paleometrics recalculated until all sites with species richness of less than five were excluded.

## 3 RESULTS

### 3.1 Evaluating model performance for the present day

**FIGURE 3:**
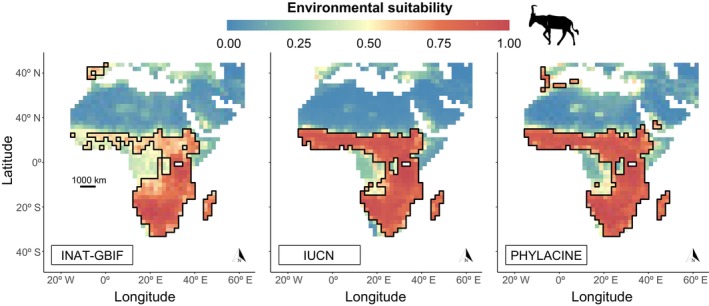
Projections of hartebeest's environmental suitability in the present day for the three occurrence datasets using BART. Environmental suitability scores vary from 0 (unsuitable) to 1 (ideal conditions). The areas delimited by the black line indicate suitable habitats above the model's optimal threshold.

Projected environmental suitability estimates based on modern climate were generally similar for all the distribution models generated; high variation in certain geographical areas was mainly dependent on the occurrence dataset used and, to a lesser extent, on the algorithm applied (environmental suitability maps averaged for all BART models for each occurrence dataset shown in Figure 3). Notable differences among occurrence datasets were seen in large areas of southern and western Africa. Moreover, the INAT‐GBIF and PHYLACINE projections showed potentially suitable environments in the Iberian Peninsula, while the IUCN map did not. Only the PHYLACINE projections showed certain areas of northern Africa as potentially suitable.

**FIGURE 4:**
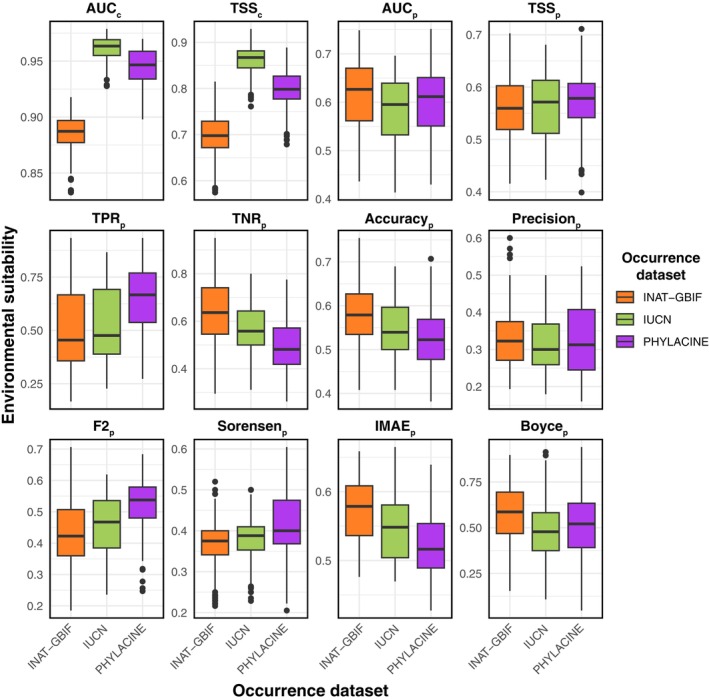
Comparison of AUC_c_, TSS_c_, and selected paleoevaluation metrics for the three occurrence datasets used in this study.

Comparing model performance across occurrence datasets, significant differences were found (evaluated using AUC and TSS; Kruskal–Wallis, *p* < .001; Figure 4 and Tables S2 and S6). The models generated with the IUCN dataset had the highest scores (AUC = mean of 0.96 ± standard deviation of 0.01; TSS = 0.86 ± 0.03), followed by PHYLACINE (AUC = 0.94 ± 0.02; TSS = 0.80 ± 0.05) and INAT‐GBIF (AUC = 0.88 ± 0.02; TSS = 0.70 ± 0.05). Comparing the modelling algorithms, the AUC scores were similar, but there were notable differences in TSS. Tree classifiers BART (TSS = 0.86 ± 0.05) and RF (TSS = 0.82 ± 0.07) slightly outperformed the others, while GBM showed significantly lower performance (TSS = 0.70 ± 0.08; Dunn: *p* < .001).

### 3.2 Evaluating model performance for hindcasts

#### 3.2.1 Presence‐absence datasets

**FIGURE 5:**
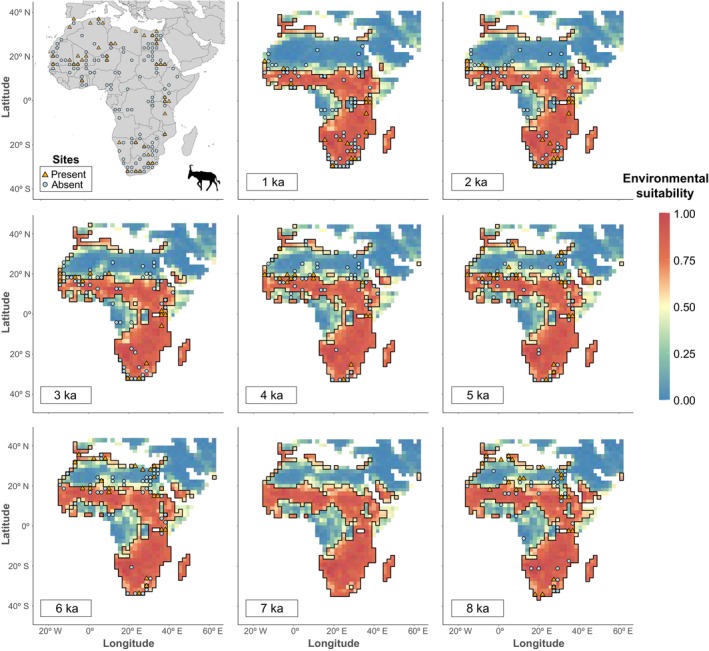
Projections of hartebeest's environmental suitability for each of the eight time slices using the PHYLACINE dataset and MaxEnt. Also shown is a map of the zooarchaeological localities from which presence and absence of hartebeest remains were used to calculate the paleometrics. Environmental suitability scores vary from 0 (unsuitable) to 1 (ideal conditions). The areas delimited by the black lines indicate suitable habitats above the model's optimal threshold. The orange triangles indicate the location of a site with identified hartebeest remains in that time slice and the blue circles indicate absence. Note that the northern expansion of suitable habitats into the Sahara is coincident with the appearance of zooarchaeological remains in that area.

Comparing the performance of models in predicting present‐day distributions against their predictions of environmental suitability in the past, as assessed using the paleozoological record, revealed marked differences (for illustration, environmental suitability maps generated with MaxEnt and the PHYLACINE dataset is shown in Figure 5; model choice based on favourable evaluation metrics, see below). Most of the suitable areas in southern and eastern Africa remain broadly similar throughout the Holocene, and between 5 and 8 ka, suitable habitats are also predicted in central tropical Africa, by the northern African Mediterranean coast, and on the Arabian Peninsula, although these areas contract over time.

When comparing paleometrics which focus on correctly predicting presences (Figure 4 and Tables S3 and S6), the PHYLACINE dataset (TPR_p_ = 0.65 ± 0.15; Sorensen_p_ = 0.42 ± 0.07; F2p = 0.53 ± 0.08) was significantly better (Kruskal–Wallis: *p* < .001; Dunn: *p* < .001) than both IUCN (TPR_p_ = 0.55 ± 0.18; Sorensen_p_ = 0.38 ± 0.05; F2_p_ = 0.46 ± 0.09) and INAT‐GBIF (TPR_p_ = 0.49 ± 0.19; Sorensen_p_ = 0.37 ± 0.05; F2_p_ = 0.42 ± 0.11). Conversely, when comparing the ability of models generated with different datasets to predict absences (Figure 4), the INAT‐GBIF dataset (TNR_p_ = 0.63 ± 0.13) was significantly better (Kruskal–Wallis: *p* < .001; Dunn: *p* < .001) than both IUCN (TNR_p_ = 0.57 ± 0.12) and the PHYLACINE dataset (TNR_p_ = 0.50 ± 0.11).

When comparing metrics that balance the models' ability to correctly predict presences and absences (Figure 4 and Tables S4 and S6), TSS_p_ values were closely matched across all datasets, with PHYLACINE (TSS_p_ = 0.57 ± 0.05) slightly outperforming both INAT‐GBIF (TSS_p_ = 0.56 ± 0.05) and IUCN (TSS_p_ = 0.56 ± 0.06). For AUC_p_, INAT‐GBIF (AUC_p_ = 0.61 ± 0.07) was significantly better (Kruskal–Wallis: *p* < .01; Dunn: *p* = .007) than IUCN (AUC_p_ = 0.58 ± 0.07) but not significantly different from PHYLACINE (AUC_p_ = 0.60 ± 0.07). For Boyce_p_, INAT‐GBIF (Boyce_p_ = 0.58 ± 0.16) was significantly better (Kruskal–Wallis: *p* < .01; Dunn: *p* = .007) than PHYLACINE (Boyce_p_ = 0.51 ± 0.17) and IUCN (Boyce_p_ = 0.48 ± 0.15).

When comparing the overall error of the models in both binary (Accuracy_p_ and Precision_p_) and continuous (IMAE_p_) form (Figure 4 and Tables S5 and S6), INAT‐GBIF (Accuracy_p_ = 0.58 ± 0.07; IMAE_p_ = 0.57 ± 0.04) showed significantly less error (Kruskal–Wallis: *p* < .01; Dunn: *p* = .007) compared to PHYLACINE (Accuracy_p_ = 0.53 ± 0.07; IMAE_p_ = 0.52 ± 0.05), while IUCN showed intermediate values (Accuracy_p_ = 0.55 ± 0.06; IMAE_p_ = 0.55 ± 0.05). No significant differences were found in Precision_p_ (INAT‐GBIF = 0.33 ± 0.07; IUCN = 0.32 ± 0.07; PHYLACINE = 0.33 ± 0.09).

#### 3.2.2 Modelling algorithms

**FIGURE 6:**
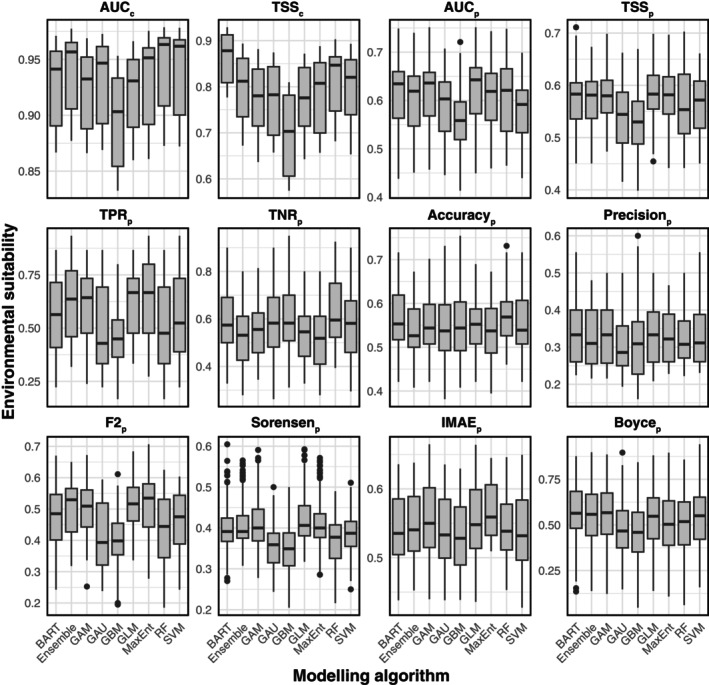
Comparison of AUC_c_, TSS_c_, and selected paleoevaluation metrics for the modelling algorithms used in this study.

The performance of models when hindcasting distributions also depended on the modelling algorithm applied. When comparing paleometrics which focus on correctly predicting presences (Figure 6 and Tables S2 and S7), the modelling algorithms with the highest values were MaxEnt (TPR_p_ = 0.63 ± 0.18; Sorensen_p_ = 0.42 ± 0.06; F2_p_ = 0.51 ± 0.09) and GLM (TPR_p_ = 0.63 ± 0.15; Sorensen_p_ = 0.41 ± 0.06; F2_p_ = 0.51 ± 0.07) and those with the lowest values were GBM (TPR_p_ = 0.46 ± 0.14; Sorensen_p_ = 0.35 ± 0.06; F2_p_ = 0.40 ± 0.08), RF (TPR_p_ = 0.50 ± 0.21; Sorensen_p_ = 0.37 ± 0.07; F2_p_ = 0.43 ± 0.13), and GAU (TPR_p_ = 0.50 ± 0.20; Sorensen_p_ = 0.35 ± 0.05; F2_p_ = 0.41 ± 0.10). Most of these differences were significantly different (see Table S2). Conversely, when comparing the ability of models generated with different modelling algorithms to predict absences (Figure 6), the highest values were shown by RF (TNR_p_ = 0.62 ± 0.13), GBM (TNR_p_ = 0.60 ± 0.13), and GAU (TNR_p_ = 0.59 ± 0.14), and the lowest by MaxEnt (TNR_p_ = 0.52 ± 0.13), GLM (TNR_p_ = 0.54 ± 0.11), and Ensemble (TNR_p_ = 0.53 ± 0.13). These differences, however, were not significant (Kruskal–Wallis: *p* > .05).

When comparing metrics that balance the models' ability to correctly predict presences and absences (Figure 6 and Tables S3, S4, and S7), TSS_p_ values were significantly higher (Kruskal–Wallis: *p* < .05; Dunn: *p* < .05) in GLM, MaxEnt, GAM, Ensemble, and BART (TSS_p_ = 0.57–0.58), than in GAU and GBM (TSSp = 0.53–0.54). AUC_p_ was significantly higher (Kruskal–Wallis: *p* < .05; Dunn: *p* < .05) in GLM and GAM (AUC_p_ = 0.62 ± 0.07) than in GBM (AUC_p_ = 0.56 ± 0.07), while Boyce_p_ was significantly higher (Kruskal–Wallis: *p* < .05; Dunn: *p* < .05) in BART (Boyce_p_ = 0.57 ± 0.15), GAM (Boyce_p_ = 0.56 ± 0.16), and Ensemble (Boyce_p_ = 0.55 ± 0.16), than in GBM (Boyce_p_ = 0.46 ± 0.16).

When comparing the overall error of the models in both binary (Accuracy_p_ and Precision_p_) and continuous (IMAE_p_) form, most modelling algorithms presented similar results and no significant differences were found (Figure 6 and Tables S6 and S7).

### 3.3 Combined presence–absence datasets and modelling algorithms

**FIGURE 7:**
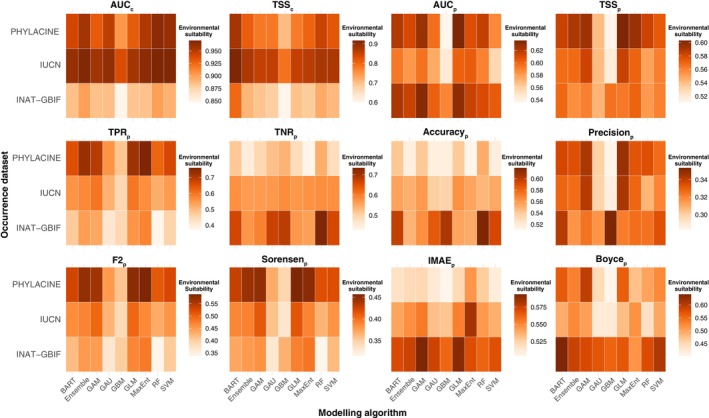
Heatmap comparing model performance across all occurrence datasets and modelling algorithms. Darker orange colours indicate higher values.

The evaluation of the projections generated using different combinations of modelling algorithms and presence‐absence datasets revealed illuminating patterns in predictive performance (Figure 7). When evaluating models for their ability to correctly predict presences in the paleozoological record (TPR_p_, F2_p_, Sorensen_p_), the best performance was achieved by the PHYLACINE dataset in combination with MaxEnt, GLM, Ensemble, and GAM, all showing average values of TPR_p_ between 0.70 and 0.76, F2_p_ between 0.56 and 0.59 and Sorensen_p_ between 0.44 and 0.46. Other modelling algorithms such as BART, SVM and RF, also performed well in combination with PHYLACINE, with average values of TPR_p_ between 0.62 and 0.67, F2_p_ between 0.51 and 0.53, and Sorensen_p_ between 0.42 and 0.43. GLM, MaxEnt, Ensemble, and GAM also performed relatively well when combined with IUCN and INAT‐GBIF (TPR_p_: 0.54–0.60; F2_p_: 0.45–0.50; Sorensen_p_: 0.38–0.41). However, GMB and GAU showed low values in these paleometrics across all datasets (TPR_p_: 0.42–0.51; F2_p_: 0.37–0.45; Sorensen_p_: 0.33–0.37). Conversely, the best models at predicting absences in the paleozoological record were those that combined the INAT‐GBIF dataset with RF, GBM, GAU, SVM, and BART, with TNR_p_ average values between 0.66 and 0.73. Most modelling algorithms combined performed similarly when combined with IUCN (TNR_p_: 0.57–0.58), and all models tended to underperform in predicting absences when combined with PHYLACINE, but especially MaxEnt, GLM, SVM, and Ensemble (TNR_p_: 0.43–0.48).

When comparing metrics that balance the correct prediction of presences and absences in the paleozoological record, the results show various differences depending on the paleometric considered. TSS_p_ average was higher in PHYLACINE datasets combined with Ensemble, GAM, GLM, and MaxEnt (TSS_p_: 0.59–0.60) and lowest in IUCN combined with GBM and GAU (TSS_p_: 0.52–0.54). AUC_p_ showed the highest average values in RF, GLM, BART, and GAM when combined with PHYLACINE and BART, GAM, and GLM, when combined with INAT'GBIF (AUC_p_: 0.62–0.64). The lowest average values of AUC_p_ were found in GBM when combined with PHYLACINE and IUCN (AUC_p_: 0.54–0.55). The highest average values of Boyce_p_ were found in BART and SVM when combined with INAT‐GBIF (Boyce_p_: 0.61–0.64), while the lowest values were shown by GBM and GAU when combined with IUCN or PHYLACINE (Boyce_p_: 0.40–0.45).

When comparing the overall error of the models in both binary (Accuracy_p_ and Precision_p_) and continuous (IMAE_p_), the results were also variable depending on the paleometrics considered. Accuracy_p_ average was higher in INAT‐GBIF when combined with BART, GBM, and RF (Accuracy_p_: 0.60–0.62) and lowest in IUCN when combined with Ensemble, GAU, GBM, MaxEnt, and SVM (Accuracy_p_: 0.51–0.52). Precision_p_ was slightly higher in INAT‐GBIF combined with BART and GBM, IUCN combined with GAM and GLM, and PHYLACINE combined with GAM, GBM, GLM (Precision_p_: 0.34–0.35), while the lowest results were shown by PHYLACINE and IUCN when combined with GBM (Precision_p_: 0.28–0.29). Finally, IMAE_p_ show the highest average vales in INAT‐GBIF combined with GAM and GLM, and IUCN combined with MaxEnt (IMAE_p_: 0.58–0.59), while PHYLACINE combined with GAU, GBM, and SVM showed the lowest values (IMAE_p_: 0.50–0.51).

### 3.4 Sensitivity analysis

**FIGURE 8:**
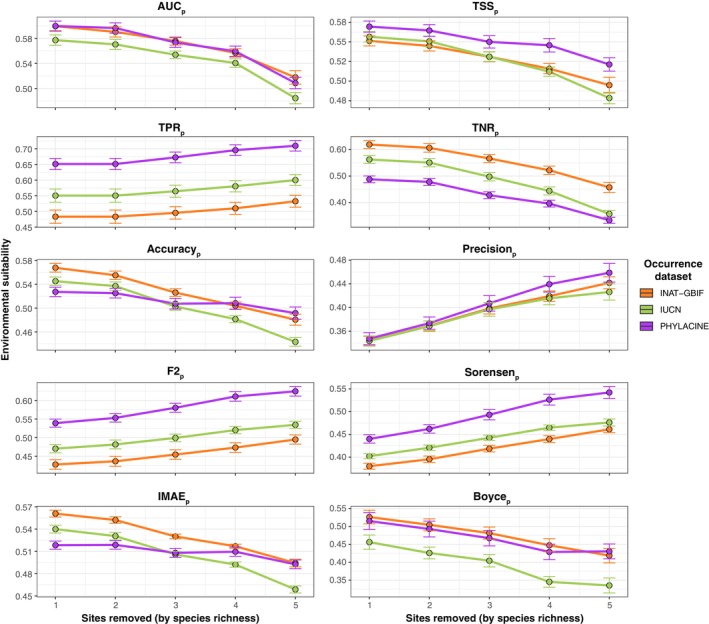
Sensitivity analysis showing the effect of paleozoological site removal on the paleometrics. The site removal is based on site species richness, whereby sites with lower species richness are progressively removed. This process continued until all sites with species richness of less than 10 are excluded.

The sensitivity analysis, where sites were removed incrementally depending on their species richness, showed that the differences between the presence‐absence datasets relative to each other were maintained despite changes in their absolute average values (Figure 8). As sites were removed, a decreasing trend was observed in paleometrics that evaluate the ability of models to predict paleozoological absences (TNR_p_) as well as those that balance the correct prediction of presences and absences (TSS_p_, AUC_p_, Boyce_p_). By contrast, paleometrics that evaluate the ability of models to predict presences (TPR_p_, F2_p_, and Sorensen_p_) tended to increase. Measures of error such as Accuracy_p_ and IMAE_p_ also show a decreasing pattern, while Precision_p_ tended to increase.

## 4 DISCUSSION

### 4.1 Occurrence data, model algorithms and the reliability of SDMs

In this study, we have compared the outputs of hartebeest distribution models using three different sources of occurrence data, i.e. data extracted from online repositories and data from two alternative range maps. Each of them has advantages and disadvantages. Our results suggest that there is a trade‐off between the correct classification of presences and the correct classification of absences in the paleozoological record. Models generated with PHYLACINE were the best at predicting presences (high values of TPR_p_, F2_p_, and Sorensen_p_) and the worst at predicting absences (low values of TNR_p_). On the contrary, models generated with INAT‐GBIF excelled at predicting absences but offered poor results at predicting presences. Finally, the models generated with IUCN ranges were the most balanced, as reflected in intermediate values across most metrics. This implies that PHYLACINE models are likely better at capturing the extent of suitable habitats in the past although they could be prone to overestimation. On the other hand, INAT‐GBIF models offer a more conservative estimates and may fail to identify the full area that the hartebeest could have inhabited in the past.

Generally, modelling algorithms did not affect the SDM outputs as much as the occurrence dataset used but there were some notable differences that require explanation. GBM generated results that underperformed and were unrealistic judging from the projections of environmental suitability (Figure S10) and the lower values of TSS_c_, AUC_c_ and paleometrics (Figure 4; Table S2). GBMs are a group of machine learning procedures that iteratively create new improved models based on previously generated ones (Natekin & Knoll, 2013). This allows the model to gradually improve its accuracy in each iteration but the approach can lead to very complex relationships between the environmental variables and the occurrence data (Elith et al., 2008), relationships that are not necessarily biologically meaningful and may generate spurious outcomes.

However, other relatively complex models, including BART and RF, demonstrated higher average AUC_c_ and TSS_c_ (Figure 4; Table S2). This is consistent with previous studies highlighting the tendency of tree‐based methods to overfit SDMs (Cutler et al., 2007; Elith et al., 2008; Li & Wang, 2013). BART and RF also presented relatively high AUC_p_ and TSSp, and these values were driven mainly by the correct prediction of absences in the paleozoological record (high values of TNR_p_). This was especially noticeable when generating models combining these two classifiers with the INAT‐GBIF dataset, which offered some of the best TNR_p_ averages but also the worst TPR_p_ values.

Similarly, more complex models, such as those produced with GAU and SVM also showed low values of TPR_p_ and relatively high values of TNR_p_, which again were accentuated when using the INAT‐GBIF dataset (Figure 4 and Figure S13). The GAU algorithm consists of a machine learning procedure using maximum likelihood estimations (MLE) applied to Gaussian distributions characterised by a mean and variance. In the context of SDMs, the mean represents the optimal value of the environmental variable(s) for the species, and the variance represents the range of values over which the species can occur (Carl & Kühn, 2007; Golding & Purse, 2016; Lichstein et al., 2002). SVM is a machine learning algorithm that allow for complex non‐linear relationships between variables, and it can often fit data better than more simple linear regression models (such as GLMs) and provide more accurate predictions of species distributions (Li & Wang, 2013). But again, models working with complex functions such as GAU and SVM can result in unrealistic ecological relationships (Hallgren et al., 2019) and we show here that they are not the best at capturing the extent of past ranges of the hartebeest in the paleozoological record.

Quadratic GLMs and GAMs (limited to two knots), are relatively simple regression models commonly used in SDMs due to their ease of interpretation and implementation. These two modelling algorithms, together with MaxEnt and the Ensemble models, when combined with the PHYLACINE dataset, produced the highest TPR_p_ values (Figure 10 and Figure S17). These four models produced also high values of AUC_p_ and TSS_p_ across all datasets. MaxEnt works by fitting a probability distribution that maximises entropy subject to constraints imposed by the environmental data and the known presences. To estimate the probability distribution, this algorithm first constructs a set of feature functions including both relatively simple functions such as linear or quadratic terms, as well as more complex functions allowing for interactions between variables (Phillips et al., 2009). This versatility explains why MaxEnt is one the most used approaches in SDM and likely the reason why it performed so well in this study. The models generated with MaxEnt and the PHYLACINE occurrence dataset had a TSS_c_ over 0.8, and they were thus good at predicting both the modern and historical distribution of the hartebeest in our study (Figure 6).

### 4.2 Integrating the zooarchaeological record with species distribution models

Our results suggest that regular evaluation metrics, such as TSS_c_ and AUC_c_, fitted to present‐day distributions may not always be ideal to select the best‐performing models, and that integrating the paleozoological record in the evaluation process by using paleometrics can significantly improve projections. However, we show that alternative paleometrics vary in their assessment of model performance. The zooarchaeological and paleontological records are usually strongly biased (Behrensmeyer et al., 2000; Lloyd et al., 2012), such that the probability of false negatives (=false absences) is likely to be much higher than that of false positives (=false presences). So which evaluation metrics should we choose and how do we select the best models?

**FIGURE 9:**
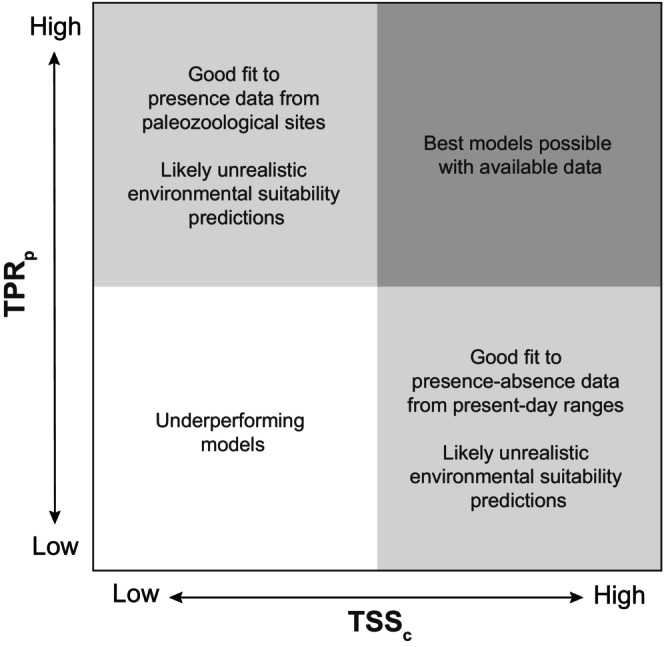
Modelling performance space comprised of TSS_c_ and TPR_p_. Models with high TSS_c_ but low TPR_p_ represent a good fit to present‐day distributions but are likely to generate unrealistic environmental suitability projections. Models with low TSS _c_ but high TPR_p_ represent a good fit to past distributions but are likely to generate unrealistic environmental suitability projections. Models with low TSS_c_ and low TPR_p_ are underperforming models, while the best models are those with high TSS_c_ and high TPR_p_ values.

**FIGURE 10:**
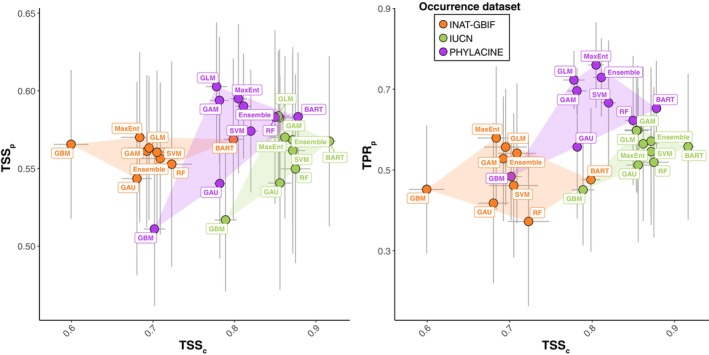
Biplots comparing TSS_c_ and the paleometrics TSS_p_ and TPR_p_ across all models, grouped and coloured by the various occurrence datasets and labelled by the different modelling algorithms used in this study. The circles represent the average across 10 replicates and the lines represent the range (minimum and maximum).

TSS_p_ balances the prediction accuracy of both presences (TPR_p_) and absences (TNR_p_). Models with high TSS_c_ but low TSS_p_ should represent a good fit to the current presence‐absence data, but these are likely to produce less realistic results when making model projections through time (Figure 9). Conversely, those models with high TSS_p_ but low TSS_c_, predict the paleozoological record better, but they may overestimate the range of suitable habitats as TSS_p_ can be significantly influenced by the uncertainty surrounding predictions of localities with unverified absences (TNR_p_). Thus, good models are those that combine both a high TSS and high TSS_p_, while models with low TSS and low TSS_p_ should be considered inadequate. For hartebeest, the PHYLACINE dataset combined with MaxEnt, GLM, GAM, or the Ensemble model offers the best results when taking into account both the present and past distribution fit (Figure 10). Paleometrics that emphasise the accurate prediction of presences may generally be more reliable indicators of model performance. These include not only TPR_p_ (how well presences are predicted) but also F2_p_ and Sorensen_p_. Unfortunately, high TPR_p_ values, especially when accompanied by low TNR_p_ values, can also result from excessive overprediction, indicating that the models might not be performing well.

As mentioned in the Introduction, when SDMs have a low capability to hindcast distributions, it may not necessarily reflect data or model limitations but could also result from shifts in the realised niche of the target species over time (Losos, 2008; Soberón, 2007; Stigall, 2012, 2014). The fundamental niche describes the environmental conditions under which a species can survive, without consideration of biotic interactions, and it may remain relatively broad and static over time. In contrast, the realised niche describes the actual environmental and biotic interactions where the species exists, and it is more dynamic due to ecological processes and altered biotic relationships (Soberón, 2010). This distinction is pivotal for SDMs, which often assume niche conservatism and do not account for changes in biotic interactions or environmental conditions that affect the realised niche (Pearman et al., 2014; Stigall et al., 2014). The issue is especially pertinent when predicting responses to novel climates lacking present‐day analogues as combinations of biotic and/or abiotic conditions that have not previously been experienced are not incorporated in the model's baseline climate and ecological settings (Soberón, 2010; Varela et al., 2009). Recent discussions by Holt et al. (2020) and Norberg et al. (2019) underscores the importance of integrating niche dynamics and external ecological pressures in SDMs where possible to enhance their accuracy and ecological relevance. However, the frequent unavailability of necessary data often limits the inclusion of these factors, as was the case in our study.

## 5 CONCLUSION

Our analysis unveiled significant variations in model performance of environmental suitability projections, both in the present and in the past, depending on the occurrence dataset used and the modelling algorithm applied. To select the most reliable modelling framework and interpret the outputs, we demonstrate the power of evaluating predicted distributions against the archaeological and fossil records of the species over extended periods of time. Although some modelling algorithms performed better than others in our study, we do not suggest that any particular model is universally the best. Thus, it is advisable to test various alternative models and choose those that perform the best to address the specific research question with the data available. Specifically, high values of traditional metrics like AUC_c_ and TSS_c_ fitted to present‐day distributions may be insufficient to guarantee that the habitat suitability projections represent realistic scenarios. We suggest adopting a combination of input data and modelling algorithms that is balanced to predict both past and present occurrences, assuming that absence data is less reliable than presence data in paleozoological records. Thus, we recommend using paleometrics such as TPR_p_, F2_p_, or Sorensen_p_, but also ensure that values for more balanced metrics such as AUC_c_, TSS_c_, AUC_p_, TSS_p_, and Boyce_p_ are reasonably high. Especially, when a model is to be used to make projections of changes in distribution in response to changing climates, we strongly advocate that its predictive power is tested through time and, given the potential for slow ecosystem responses and distributional lags, that the archaeological and paleontological records over an appropriately long time interval is used to evaluate the model performance.

## AUTHOR CONTRIBUTIONS

**Ignacio A. Lazagabaster:** Conceptualization (lead); formal analysis (lead); investigation (lead); methodology (lead); visualization (lead); writing – original draft (lead); writing – review and editing (lead). **Chris D. Thomas:** Conceptualization (lead); formal analysis (supporting); funding acquisition (supporting); investigation (equal); methodology (equal); supervision (equal); writing – original draft (equal); writing – review and editing (equal). **Juliet V. Spedding:** Conceptualization (supporting); resources (supporting); writing – review and editing (equal). **Salima Ikram:** Conceptualization (supporting); funding acquisition (supporting); methodology (supporting); validation (supporting); writing – review and editing (equal). **Irene Solano‐Regadera:** Methodology (supporting); writing – review and editing (supporting). **Steven Snape:** Conceptualization (supporting); funding acquisition (supporting); writing – review and editing (equal). **Jakob Bro‐Jørgensen:** Conceptualization (lead); formal analysis (supporting); funding acquisition (lead); investigation (equal); methodology (equal); project administration (lead); supervision (lead); writing – original draft (equal); writing – review and editing (equal).

## FUNDING INFORMATION

This work is part of the Biodiversity in Ancient Egypt during Societal Transitions (BEAST) project, funded by a Leverhulme Trust Research Project Grant with No. RPG‐2021‐104 awarded to JB‐J.

## CONFLICT OF INTEREST STATEMENT

No conflict of interest is reported.

### OPEN RESEARCH BADGES

This article has earned an Open Data badge for making publicly available the digitally‐shareable data necessary to reproduce the reported results. The data is available at https://osf.io/wck7n/?view_only=64874fd144754af28b6e4684f568bf44.

## Supporting information


Data S1:

## Data Availability

All data and code used in this work are available as Supporting Information in the online version of this manuscript and at Open Science Framework: https://osf.io/wck7n/?view_only=64874fd144754af28b6e4684f568bf44.
