# Supplementary material for: Evaluating species distribution model predictions through time against paleozoological records
Source: Ecol Evol. 2024 Oct 22;14(10):e70288. doi: 10.1002/ece3.70288 (PMC11496045; doi:10.1002/ece3.70288)
Supplement: Supplementary file 1 — Data S1: [file ECE3-14-e70288-s001.docx]

Supporting Information for:

**Evaluating species distribution model predictions through time with paleozoological records**

This supporting information includes:

| **Supp. Inf. S1.** The paleo.eval function in R……………………………………………… | 2 |
| --- | --- |
| **Tables S1-S7**……………………………………………………………………………… | 5 |
| **Figures S1-S18**……………………………………………………………………………. | 12 |

**Supp. Inf. S1. The *paleo.eval* function in R**

**Description**

Calculate paleoevaluation metrics (sensitivity_p_, specificity_p_, and TSS_p_) from species distribution model environmental suitability hindcast projections.

**Usage**

paleo.eval (raster_projections, paleo_dataset, taxon_name, taxon_colname, start_date_colname, end_date_colname, latitude_colname, longitude_colname, round_date_colname, round_date_number, threshold, time_slices, plots, messages)

**Arguments**

| raster_projections | SpatRasters: a collection of terra SpatRasters representing species distribution model environmental suitability hindcast predictions for different time slices either in continuous or binary form (if rasters are in continuous form, a threshold should be provided). The rasters’ names should match the values of time_slices. |
| --- | --- |
| paleo_dataset | data.frame: a dataset with spatial information (latitude and longitude) on paleozoological localities, species composition, and age. If start and end dates (preferably obtained from radiocarbon calibrated ages before present, calBP) are given for all records in the dataset, the function rounds the dates to the corresponding values of time_slices. For example, in 1000-yr time slices, the start date 5736 calBP and end date 3210 calBP, would then match this record to the time slices representing 3000, 4000, 5000, and 6000 years ago (ka). |
| taxon_name | character: name of the taxon analyzed. Should match a taxon name in the paleo_dataset |
| taxon_colname | character: name of the column with taxon names in paleo_dataset |
| start_date_colname | character: name of the column with start dates (records’ oldest dates) in paleo_dataset |
| end_date_colname | character: name of the column with end dates (records’ youngest dates) in paleo_dataset |
| latitude_colname | character: name of the column with latitude coordinates in paleo_dataset. The coordinate reference system should match that of raster_projections |
| longitude_colname | character: name of the column with longitude coordinates in paleo_dataset. The coordinate reference system should match that of raster_projections |
| round_date_colname | character: name of the column with rounded dates or time periods matching the values of time_slices |
| round_date_number | numeric: If round_date_colname is not provided and time_slices are numbers, then a round number to which approximate the dates should be given. For example, if round_date_number=100, the start date 5736 calBP and end date 5410 calBP, would then match this record to the time slices representing 5800, 5600, 5500, and 5400 years ago (ka). |
| threshold | numeric: a threshold value obtained from the species distribution modelling output |
| time_slices | character vector: names of the rounded dates or time periods matching the names of raster_projections |
| plots | boolean: if TRUE, plots are returned showing hindcast projections with matched paleozoological sites with presences and absences of the chosen taxon. if FALSE, plots are not returned. Default is TRUE. |
| messages | boolean: if TRUE, messages are returned. if FALSE, no messages are printed. Default is TRUE. |

**Details**

The function follows the confusion matrix and formulae described in [Allouche et al. (2006)](https://paperpile.com/c/PyavUR/GqNh), but using paleozoological records as validation sets.

**Value**

A list of data.frames with coordinates of paleozoological localities with presence and absence of taxon analyzed for each time slice

A data.frame summarizing the paleoevaluation metrics for each time slice

A ggplot showing all the paleozoological localities with presence and absence of the chosen taxon

A series of ggplots showing the paleozoological localities with presence and absence of the chosen taxon for each time slice

**Author**

Written by xxx

**Function references**

[Allouche, O., Tsoar, A., & Kadmon, R. (2006). Assessing the accuracy of species distribution models: prevalence, kappa and the true skill statistic (TSS). *The Journal of Applied Ecology*, *43*(6), 1223–1232. https://doi.org/](http://paperpile.com/b/PyavUR/GqNh)[10.1111/j.1365-2664.2006.01214.x](http://dx.doi.org/10.1111/j.1365-2664.2006.01214.x)

**Table S1.** Summary of the main custom functions used in the species distribution modelling and the most relevant library dependencies.

| Function name | Goal | Function  dependency | Library  dependency |
| --- | --- | --- | --- |
| get_occurrences_INAT_GBIF  get_occurrences_IUCN  get_occurrences_PHYL | Generate occurrence data | get_inat_obs  occ_search  clean_coordinates  gridRecords | rinat [(Barve et al., 2022)](https://paperpile.com/c/PyavUR/F9Iw)  rgbif [(Chamberlain et al., 2022)](https://paperpile.com/c/PyavUR/qCA6)  CoordinateCleaner [(Zizka et al., 2019)](https://paperpile.com/c/PyavUR/iWJV)  fuzzySim [(Barbosa, 2015)](https://paperpile.com/c/PyavUR/N2CB) |
| get_absences_INAT_GBIF  get_absences_IUCN  get_absences_PHYL | Generate absence data | sample_pseudoabs  sample_background  sdm_extract | flexsdm [(Velazco et al., 2022)](https://paperpile.com/c/PyavUR/CJfi)  flexsdm [(Velazco et al., 2022)](https://paperpile.com/c/PyavUR/CJfi)  flexsdm [(Velazco et al., 2022)](https://paperpile.com/c/PyavUR/CJfi) |
| select_variables | Variable selection | correct_colinvar  varimp.diag  variable.step | flexsdm [(Velazco et al., 2022)](https://paperpile.com/c/PyavUR/CJfi)  embarcadero [(Carlson, 2020)](https://paperpile.com/c/PyavUR/Y0Gu)  embarcadero [(Carlson, 2020)](https://paperpile.com/c/PyavUR/Y0Gu) |
| BART_model | BART models | bart  threshMeasures  part_random  partial  predict  predict_bart_df | embarcadero [(Carlson, 2020)](https://paperpile.com/c/PyavUR/Y0Gu)  modEvA [(Barbosa et al., 2016)](https://paperpile.com/c/PyavUR/hZnf)  flexsdm [(Velazco et al., 2022)](https://paperpile.com/c/PyavUR/CJfi)  embarcadero [(Carlson, 2020)](https://paperpile.com/c/PyavUR/Y0Gu)  terra [(Hijmans et al., 2022)](https://paperpile.com/c/PyavUR/jCPp)  https://github.com/AMBarbosa/unpackaged |
| SDMflex_model | SDMflex  models | fit_glm  fit_gam  fit_gau  fit_raf  fit_max  fit_gbm  fit_svm  fit_ensemble  sdm_summarize  sdm_predict  data_pdp | flexsdm [(Velazco et al., 2022)](https://paperpile.com/c/PyavUR/CJfi)  flexsdm [(Velazco et al., 2022)](https://paperpile.com/c/PyavUR/CJfi)  flexsdm [(Velazco et al., 2022)](https://paperpile.com/c/PyavUR/CJfi)  flexsdm [(Velazco et al., 2022)](https://paperpile.com/c/PyavUR/CJfi)  flexsdm [(Velazco et al., 2022)](https://paperpile.com/c/PyavUR/CJfi)  flexsdm [(Velazco et al., 2022)](https://paperpile.com/c/PyavUR/CJfi)  flexsdm [(Velazco et al., 2022)](https://paperpile.com/c/PyavUR/CJfi)  flexsdm [(Velazco et al., 2022)](https://paperpile.com/c/PyavUR/CJfi)  flexsdm [(Velazco et al., 2022)](https://paperpile.com/c/PyavUR/CJfi)  flexsdm [(Velazco et al., 2022)](https://paperpile.com/c/PyavUR/CJfi)  flexsdm [(Velazco et al., 2022)](https://paperpile.com/c/PyavUR/CJfi) |

**Table S2.** Summary statistics of evaluation metrics for each occurrence dataset and modelling algorithm used in this study. Each row summarizes 10 different presence-absence replicates. Abbreviations: TSS_c_ = True Skill Statistic calculated for current climatic conditions; AUC_c_, Area Under the Curve calculated for current climatic conditions; sd, standard deviation.

| **Occurrence dataset** | **Modelling algorithm** | **AUC_c_ mean** | **AUC_c_ sd** | **TSS_c_ mean** | **TSS_c_ sd** |
| --- | --- | --- | --- | --- | --- |
| INAT-GBIF | BART | 0.88 | 0.01 | 0.80 | 0.01 |
| INAT-GBIF | Ensemble | 0.90 | 0.01 | 0.71 | 0.02 |
| INAT-GBIF | GAM | 0.88 | 0.01 | 0.69 | 0.03 |
| INAT-GBIF | GAU | 0.89 | 0.01 | 0.68 | 0.02 |
| INAT-GBIF | GBM | 0.85 | 0.01 | 0.60 | 0.02 |
| INAT-GBIF | GLM | 0.88 | 0.01 | 0.70 | 0.03 |
| INAT-GBIF | MaxEnt | 0.88 | 0.01 | 0.68 | 0.02 |
| INAT-GBIF | RF | 0.90 | 0.01 | 0.72 | 0.03 |
| INAT-GBIF | SVM | 0.89 | 0.01 | 0.70 | 0.03 |
| IUCN | BART | 0.96 | 0.01 | 0.92 | 0.01 |
| IUCN | Ensemble | 0.97 | 0.01 | 0.87 | 0.02 |
| IUCN | GAM | 0.96 | 0.01 | 0.86 | 0.02 |
| IUCN | GAU | 0.96 | 0.01 | 0.86 | 0.01 |
| IUCN | GBM | 0.94 | 0.01 | 0.79 | 0.01 |
| IUCN | GLM | 0.96 | 0.01 | 0.85 | 0.01 |
| IUCN | MaxEnt | 0.97 | 0.01 | 0.86 | 0.02 |
| IUCN | RF | 0.97 | 0.00 | 0.87 | 0.02 |
| IUCN | SVM | 0.97 | 0.01 | 0.87 | 0.02 |
| PHYL | BART | 0.94 | 0.00 | 0.88 | 0.01 |
| PHYL | Ensemble | 0.96 | 0.00 | 0.81 | 0.01 |
| PHYL | GAM | 0.93 | 0.01 | 0.78 | 0.01 |
| PHYL | GAU | 0.95 | 0.00 | 0.78 | 0.01 |
| PHYL | GBM | 0.90 | 0.00 | 0.70 | 0.01 |
| PHYL | GLM | 0.93 | 0.01 | 0.78 | 0.01 |
| PHYL | MaxEnt | 0.95 | 0.00 | 0.80 | 0.01 |
| PHYL | RF | 0.96 | 0.00 | 0.85 | 0.01 |
| PHYL | SVM | 0.96 | 0.00 | 0.82 | 0.01 |

**Table S3.** Summary statistics of evaluation paleometrics for each occurrence dataset and modelling algorithm used in this study. Each row summarizes 10 different presence-absence replicates. Abbreviations: TPR_p_ = True Positive Rate; TNR_p_, True Negative Rate; sd, standard deviation.

| **Occurrence dataset** | **Modelling algorithm** | **TPR_p_ mean** | **TPR_p_ sd** | **F2_p_ mean** | **F2_p_ sd** | **Sorensen_p_ mean** | **Sorensen_p_ sd** | **TNR_p_ mean** | **TNR_p_ sd** |
| --- | --- | --- | --- | --- | --- | --- | --- | --- | --- |
| INAT-GBIF | BART | 0.48 | 0.18 | 0.42 | 0.10 | 0.37 | 0.05 | 0.66 | 0.12 |
| INAT-GBIF | Ensemble | 0.54 | 0.17 | 0.45 | 0.08 | 0.38 | 0.03 | 0.57 | 0.12 |
| INAT-GBIF | GAM | 0.53 | 0.15 | 0.45 | 0.07 | 0.38 | 0.03 | 0.59 | 0.11 |
| INAT-GBIF | GAU | 0.42 | 0.20 | 0.37 | 0.11 | 0.33 | 0.05 | 0.67 | 0.14 |
| INAT-GBIF | GBM | 0.45 | 0.16 | 0.40 | 0.09 | 0.37 | 0.06 | 0.68 | 0.14 |
| INAT-GBIF | GLM | 0.56 | 0.14 | 0.47 | 0.06 | 0.39 | 0.03 | 0.57 | 0.10 |
| INAT-GBIF | MaxEnt | 0.58 | 0.18 | 0.48 | 0.08 | 0.40 | 0.03 | 0.56 | 0.12 |
| INAT-GBIF | RF | 0.37 | 0.21 | 0.34 | 0.14 | 0.31 | 0.07 | 0.73 | 0.11 |
| INAT-GBIF | SVM | 0.46 | 0.18 | 0.41 | 0.10 | 0.36 | 0.04 | 0.66 | 0.12 |
| IUCN | BART | 0.56 | 0.18 | 0.46 | 0.09 | 0.39 | 0.04 | 0.58 | 0.12 |
| IUCN | Ensemble | 0.57 | 0.18 | 0.47 | 0.08 | 0.39 | 0.04 | 0.57 | 0.12 |
| IUCN | GAM | 0.60 | 0.16 | 0.50 | 0.06 | 0.41 | 0.03 | 0.57 | 0.12 |
| IUCN | GAU | 0.51 | 0.19 | 0.43 | 0.10 | 0.36 | 0.05 | 0.57 | 0.11 |
| IUCN | GBM | 0.45 | 0.14 | 0.39 | 0.07 | 0.33 | 0.05 | 0.58 | 0.11 |
| IUCN | GLM | 0.60 | 0.15 | 0.50 | 0.06 | 0.41 | 0.03 | 0.57 | 0.12 |
| IUCN | MaxEnt | 0.56 | 0.19 | 0.47 | 0.09 | 0.39 | 0.04 | 0.58 | 0.13 |
| IUCN | RF | 0.52 | 0.19 | 0.43 | 0.09 | 0.37 | 0.03 | 0.58 | 0.10 |
| IUCN | SVM | 0.55 | 0.18 | 0.46 | 0.09 | 0.38 | 0.04 | 0.58 | 0.11 |
| PHYL | BART | 0.65 | 0.12 | 0.53 | 0.05 | 0.43 | 0.06 | 0.51 | 0.11 |
| PHYL | Ensemble | 0.73 | 0.10 | 0.57 | 0.03 | 0.44 | 0.07 | 0.45 | 0.10 |
| PHYL | GAM | 0.70 | 0.06 | 0.56 | 0.05 | 0.45 | 0.09 | 0.49 | 0.10 |
| PHYL | GAU | 0.56 | 0.18 | 0.45 | 0.08 | 0.37 | 0.04 | 0.52 | 0.12 |
| PHYL | GBM | 0.48 | 0.11 | 0.41 | 0.07 | 0.34 | 0.07 | 0.54 | 0.09 |
| PHYL | GLM | 0.72 | 0.07 | 0.58 | 0.05 | 0.46 | 0.08 | 0.48 | 0.10 |
| PHYL | MaxEnt | 0.76 | 0.11 | 0.59 | 0.05 | 0.45 | 0.07 | 0.43 | 0.09 |
| PHYL | RF | 0.62 | 0.16 | 0.51 | 0.06 | 0.42 | 0.03 | 0.54 | 0.10 |
| PHYL | SVM | 0.67 | 0.15 | 0.53 | 0.05 | 0.42 | 0.05 | 0.48 | 0.13 |

**Table S4.** Summary statistics of evaluation paleometrics for each occurrence dataset and modelling algorithm used in this study. Each row summarizes 10 different presence-absence replicates. Abbreviations: TSS_p_ = True Skill Statistic; AUC_p_, Area Under the Curve; sd, standard deviation.

| **Occurrence dataset** | **Modelling algorithm** | **TSS_p_ mean** | **TSS_p_ sd** | **AUC_p_ mean** | **AUC_p_ sd** | **Boyce_p_ mean** | **Boyce_p_ sd** |
| --- | --- | --- | --- | --- | --- | --- | --- |
| INAT-GBIF | BART | 0.57 | 0.05 | 0.62 | 0.07 | 0.64 | 0.13 |
| INAT-GBIF | Ensemble | 0.56 | 0.05 | 0.61 | 0.07 | 0.59 | 0.14 |
| INAT-GBIF | GAM | 0.56 | 0.05 | 0.63 | 0.07 | 0.58 | 0.16 |
| INAT-GBIF | GAU | 0.54 | 0.06 | 0.60 | 0.07 | 0.57 | 0.16 |
| INAT-GBIF | GBM | 0.57 | 0.05 | 0.59 | 0.07 | 0.56 | 0.16 |
| INAT-GBIF | GLM | 0.56 | 0.05 | 0.63 | 0.07 | 0.57 | 0.17 |
| INAT-GBIF | MaxEnt | 0.57 | 0.05 | 0.61 | 0.08 | 0.52 | 0.15 |
| INAT-GBIF | RF | 0.55 | 0.07 | 0.61 | 0.07 | 0.58 | 0.14 |
| INAT-GBIF | SVM | 0.56 | 0.05 | 0.60 | 0.06 | 0.61 | 0.16 |
| IUCN | BART | 0.57 | 0.05 | 0.59 | 0.07 | 0.49 | 0.13 |
| IUCN | Ensemble | 0.57 | 0.05 | 0.58 | 0.07 | 0.52 | 0.15 |
| IUCN | GAM | 0.58 | 0.04 | 0.60 | 0.07 | 0.51 | 0.16 |
| IUCN | GAU | 0.54 | 0.07 | 0.58 | 0.07 | 0.42 | 0.11 |
| IUCN | GBM | 0.52 | 0.05 | 0.55 | 0.06 | 0.43 | 0.12 |
| IUCN | GLM | 0.58 | 0.04 | 0.60 | 0.07 | 0.48 | 0.16 |
| IUCN | MaxEnt | 0.57 | 0.05 | 0.60 | 0.07 | 0.52 | 0.15 |
| IUCN | RF | 0.55 | 0.06 | 0.59 | 0.07 | 0.44 | 0.15 |
| IUCN | SVM | 0.56 | 0.07 | 0.56 | 0.06 | 0.50 | 0.17 |
| PHYL | BART | 0.58 | 0.04 | 0.62 | 0.07 | 0.57 | 0.16 |
| PHYL | Ensemble | 0.59 | 0.03 | 0.61 | 0.06 | 0.53 | 0.17 |
| PHYL | GAM | 0.59 | 0.04 | 0.63 | 0.06 | 0.60 | 0.15 |
| PHYL | GAU | 0.54 | 0.05 | 0.60 | 0.06 | 0.45 | 0.14 |
| PHYL | GBM | 0.51 | 0.05 | 0.54 | 0.05 | 0.40 | 0.15 |
| PHYL | GLM | 0.60 | 0.04 | 0.64 | 0.06 | 0.56 | 0.14 |
| PHYL | MaxEnt | 0.59 | 0.05 | 0.61 | 0.06 | 0.48 | 0.16 |
| PHYL | RF | 0.58 | 0.06 | 0.62 | 0.07 | 0.50 | 0.17 |
| PHYL | SVM | 0.57 | 0.04 | 0.59 | 0.06 | 0.52 | 0.16 |

**Table S5.** Summary statistics of evaluation paleometrics for each occurrence dataset and modelling algorithm used in this study. Each row summarizes 10 different presence-absence replicates. Abbreviations: IMAE_p_ = Inverse Mean Absolute Error; sd, standard deviation.

| **Occurrence dataset** | **Modelling algorithm** | **Accuracy_p_ mean** | **Accuracy_p_ sd** | **Precision_p_ mean** | **Precision_p_ sd** | **IMAE_p_ mean** | **IMAE_p_ sd** |
| --- | --- | --- | --- | --- | --- | --- | --- |
| INAT-GBIF | BART | 0.60 | 0.06 | 0.34 | 0.07 | 0.57 | 0.04 |
| INAT-GBIF | Ensemble | 0.55 | 0.06 | 0.32 | 0.06 | 0.57 | 0.04 |
| INAT-GBIF | GAM | 0.56 | 0.06 | 0.32 | 0.06 | 0.59 | 0.04 |
| INAT-GBIF | GAU | 0.59 | 0.07 | 0.32 | 0.07 | 0.57 | 0.04 |
| INAT-GBIF | GBM | 0.60 | 0.08 | 0.35 | 0.09 | 0.56 | 0.04 |
| INAT-GBIF | GLM | 0.55 | 0.05 | 0.32 | 0.06 | 0.59 | 0.04 |
| INAT-GBIF | MaxEnt | 0.55 | 0.06 | 0.33 | 0.06 | 0.57 | 0.04 |
| INAT-GBIF | RF | 0.62 | 0.06 | 0.32 | 0.05 | 0.56 | 0.04 |
| INAT-GBIF | SVM | 0.59 | 0.06 | 0.33 | 0.07 | 0.57 | 0.04 |
| IUCN | BART | 0.56 | 0.06 | 0.33 | 0.08 | 0.54 | 0.05 |
| IUCN | Ensemble | 0.55 | 0.06 | 0.33 | 0.07 | 0.55 | 0.04 |
| IUCN | GAM | 0.56 | 0.06 | 0.34 | 0.08 | 0.55 | 0.05 |
| IUCN | GAU | 0.54 | 0.05 | 0.30 | 0.06 | 0.53 | 0.04 |
| IUCN | GBM | 0.54 | 0.06 | 0.29 | 0.07 | 0.53 | 0.04 |
| IUCN | GLM | 0.56 | 0.06 | 0.34 | 0.08 | 0.55 | 0.05 |
| IUCN | MaxEnt | 0.56 | 0.06 | 0.33 | 0.07 | 0.58 | 0.04 |
| IUCN | RF | 0.55 | 0.05 | 0.31 | 0.05 | 0.54 | 0.04 |
| IUCN | SVM | 0.55 | 0.06 | 0.32 | 0.07 | 0.54 | 0.05 |
| PHYL | BART | 0.54 | 0.07 | 0.34 | 0.09 | 0.52 | 0.05 |
| PHYL | Ensemble | 0.52 | 0.07 | 0.33 | 0.09 | 0.52 | 0.05 |
| PHYL | GAM | 0.54 | 0.07 | 0.34 | 0.10 | 0.52 | 0.06 |
| PHYL | GAU | 0.52 | 0.06 | 0.30 | 0.07 | 0.51 | 0.05 |
| PHYL | GBM | 0.52 | 0.06 | 0.28 | 0.08 | 0.50 | 0.04 |
| PHYL | GLM | 0.54 | 0.07 | 0.35 | 0.10 | 0.52 | 0.06 |
| PHYL | MaxEnt | 0.51 | 0.07 | 0.33 | 0.08 | 0.55 | 0.03 |
| PHYL | RF | 0.55 | 0.05 | 0.33 | 0.07 | 0.53 | 0.04 |
| PHYL | SVM | 0.52 | 0.07 | 0.33 | 0.08 | 0.51 | 0.06 |

**Table S6.** Results of non-parametric Dunn tests comparing different occurrence datasets.

| **Metric** | **Comparison** | **Z-score** | **p-value** |
| --- | --- | --- | --- |
| **Current** | | | |
| AUC_c_ | INAT-GBIF - IUCN | -12.72 | 0.0000 |
| AUC_c_ | INAT-GBIF - PHYL | -8.03 | 0.0000 |
| AUC_c_ | IUCN - PHYL | 4.70 | 0.0000 |
| TSS_c_ | INAT-GBIF - IUCN | -12.04 | 0.0000 |
| TSS_c_ | INAT-GBIF - PHYL | -6.66 | 0.0000 |
| TSS_c_ | IUCN - PHYL | 5.38 | 0.0000 |
| **Presences** | | | |
| TPR_p_ | INAT-GBIF - PHYL | -5.67 | 0.0000 |
| TPR_p_ | IUCN - PHYL | -3.73 | 0.0003 |
| F2_p_ | INAT-GBIF - PHYL | -6.26 | 0.0000 |
| F2_p_ | IUCN - PHYL | -4.15 | 0.0001 |
| Sorensen_p_ | INAT-GBIF - IUCN | -2.26 | 0.0354 |
| Sorensen_p_ | INAT-GBIF - PHYL | -5.77 | 0.0000 |
| Sorensen_p_ | IUCN - PHYL | -3.51 | 0.0007 |
| **Absences** | | | |
| TNR_p_ | INAT-GBIF - IUCN | 2.67 | 0.0112 |
| TNR_p_ | INAT-GBIF - PHYL | 6.80 | 0.0000 |
| TNR_p_ | IUCN - PHYL | 4.12 | 0.0001 |
| **Balanced** | | | |
| AUC_p_ | INAT-GBIF - IUCN | 2.81 | 0.0074 |
| BOYCE_p_ | INAT-GBIF - IUCN | 5.77 | 0.0000 |
| BOYCE_p_ | INAT-GBIF - PHYL | 4.05 | 0.0001 |
| **Error** | | | |
| Accuracy_p_ | INAT-GBIF - PHYL | 3.35 | 0.0012 |
| IMAE_p_ | INAT-GBIF - PHYL | 4.14 | 0.0001 |
| IMAE_p_ | IUCN - PHYL | 2.39 | 0.0256 |

**Table S7.** Results of non-parametric Dunn tests comparing different modelling algorithms. Only significant comparisons are shown.

| **Metric** | **Comparison** | **Z-score** | **p-value** |  | **Metric** | **Comparison** | **Z-score** | **p-value** |
| --- | --- | --- | --- | --- | --- | --- | --- | --- |
| **Current** | | | |  | **Presences (continuation)** | | | |
| TSS_c_ | BART - GBM | 6.95 | 0.0000 |  | Sorensenp | GAM - GBM | 4.90 | 0.0000 |
| TSS_c_ | BART - GLM | 4.88 | 0.0000 |  | Sorensenp | Ensemble - GBM | 4.52 | 0.0001 |
| TSS_c_ | BART - GAM | 4.73 | 0.0000 |  | Sorensenp | GAU - GLM | -4.35 | 0.0003 |
| TSS_c_ | BART - GAU | 4.73 | 0.0000 |  | Sorensenp | GAU - MaxEnt | -4.05 | 0.0009 |
| TSS_c_ | GBM - RF | -4.73 | 0.0000 |  | Sorensenp | GAM - GAU | 3.89 | 0.0018 |
| TSS_c_ | GBM - SVM | -4.14 | 0.0006 |  | Sorensenp | BART - GBM | 3.80 | 0.0026 |
| TSS_c_ | Ensemble - GBM | 3.99 | 0.0012 |  | Sorensenp | Ensemble - GAU | 3.51 | 0.0082 |
| TSS_c_ | BART - MaxEnt | 3.99 | 0.0012 |  | Sorensenp | GBM - SVM | -3.33 | 0.0156 |
| AUC_c_ | GBM - RF | -5.03 | 0.0000 |  | **Balanced** | | | |
| AUC_c_ | GBM - SVM | -4.29 | 0.0003 |  | TSSc | BART - GBM | 6.95 | 0.0000 |
| AUC_c_ | Ensemble - GBM | 4.14 | 0.0006 |  | TSSc | BART - GLM | 4.88 | 0.0000 |
| AUC_c_ | GLM - RF | -4.14 | 0.0006 |  | TSSc | BART - GAM | 4.73 | 0.0000 |
| AUC_c_ | GAM - RF | -3.40 | 0.0121 |  | TSSc | BART - GAU | 4.73 | 0.0000 |
| AUC_c_ | GLM - SVM | -3.40 | 0.0121 |  | TSSc | GBM - RF | -4.73 | 0.0000 |
| AUC_c_ | Ensemble - GLM | 3.25 | 0.0205 |  | TSSc | GBM - SVM | -4.14 | 0.0006 |
| **Presences** | | | |  | TSSc | Ensemble - GBM | 3.99 | 0.0012 |
| TPRp | GBM - MaxEnt | -3.35 | 0.0145 |  | TSSc | BART - MaxEnt | 3.99 | 0.0012 |
| TPRp | GBM - GLM | -3.33 | 0.0157 |  | TSSp | GBM - GLM | -4.09 | 0.0008 |
| F2p | GBM - MaxEnt | -4.04 | 0.0010 |  | TSSp | GAM - GBM | 3.74 | 0.0034 |
| F2p | GBM - GLM | -4.03 | 0.0010 |  | TSSp | GBM - MaxEnt | -3.73 | 0.0034 |
| F2p | Ensemble - GBM | 3.57 | 0.0063 |  | TSSp | Ensemble - GBM | 3.45 | 0.0101 |
| F2p | GAM - GBM | 3.47 | 0.0095 |  | TSSp | BART - GBM | 3.42 | 0.0114 |
| F2p | GAU - MaxEnt | -3.34 | 0.0151 |  | AUCp | GBM - GLM | -3.38 | 0.0129 |
| F2p | GAU - GLM | -3.33 | 0.0158 |  | AUCp | GAM - GBM | 3.02 | 0.0449 |
| Sorensenp | GBM - GLM | -5.36 | 0.0000 |  | BOYCEp | BART - GBM | 4.08 | 0.0008 |
| Sorensenp | GBM - MaxEnt | -5.07 | 0.0000 |  | BOYCEp | GBM - GLM | -3.20 | 0.0245 |


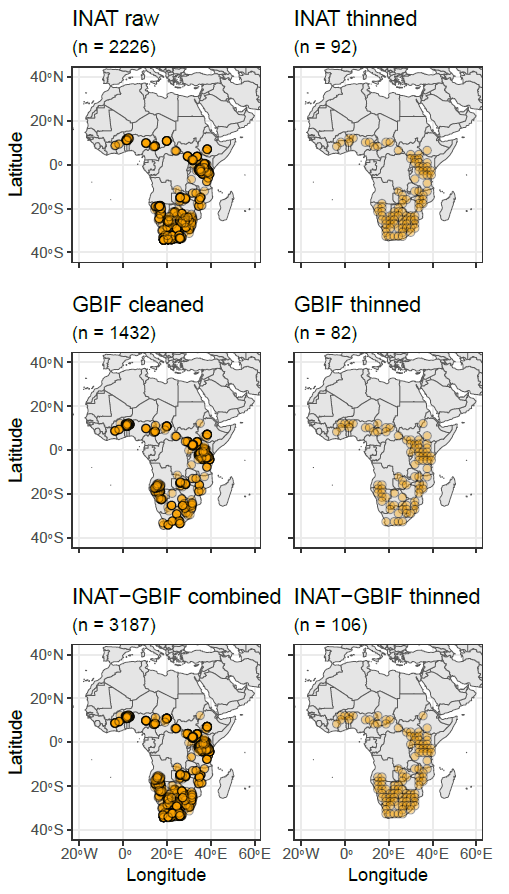


**Figure S1.** Occurrences downloaded from GBIF and iNaturalist, before and after cleaning and thinning. The combined resultant dataset has 106 occurrences (right lowermost panel).


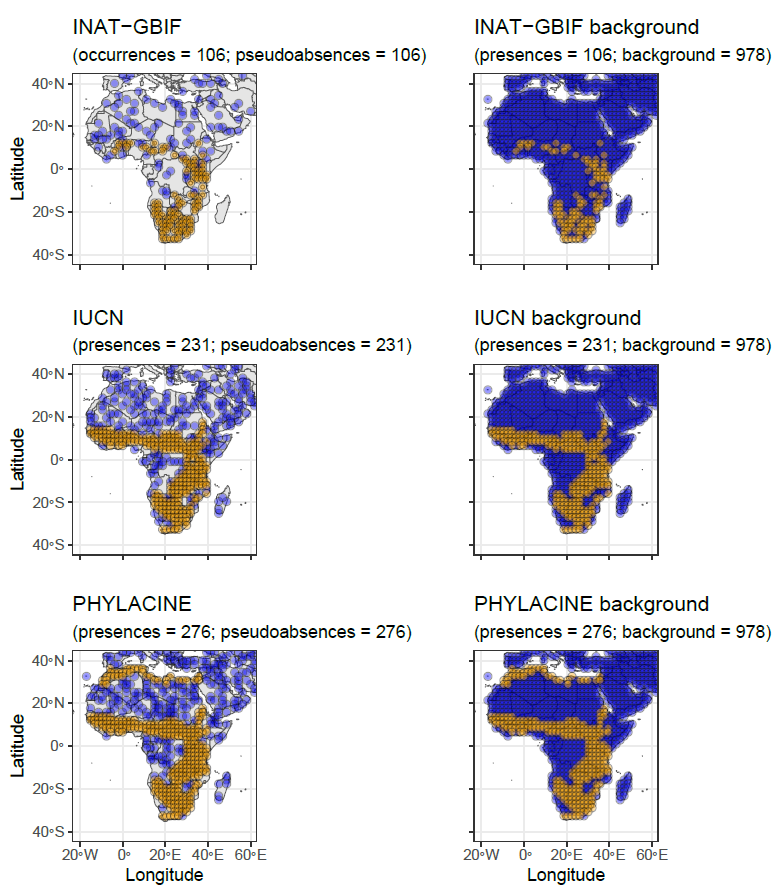


**Figure S2.** Example of one of the 10 presence - (pseudo) absence data points generated for each of the occurrence datasets.


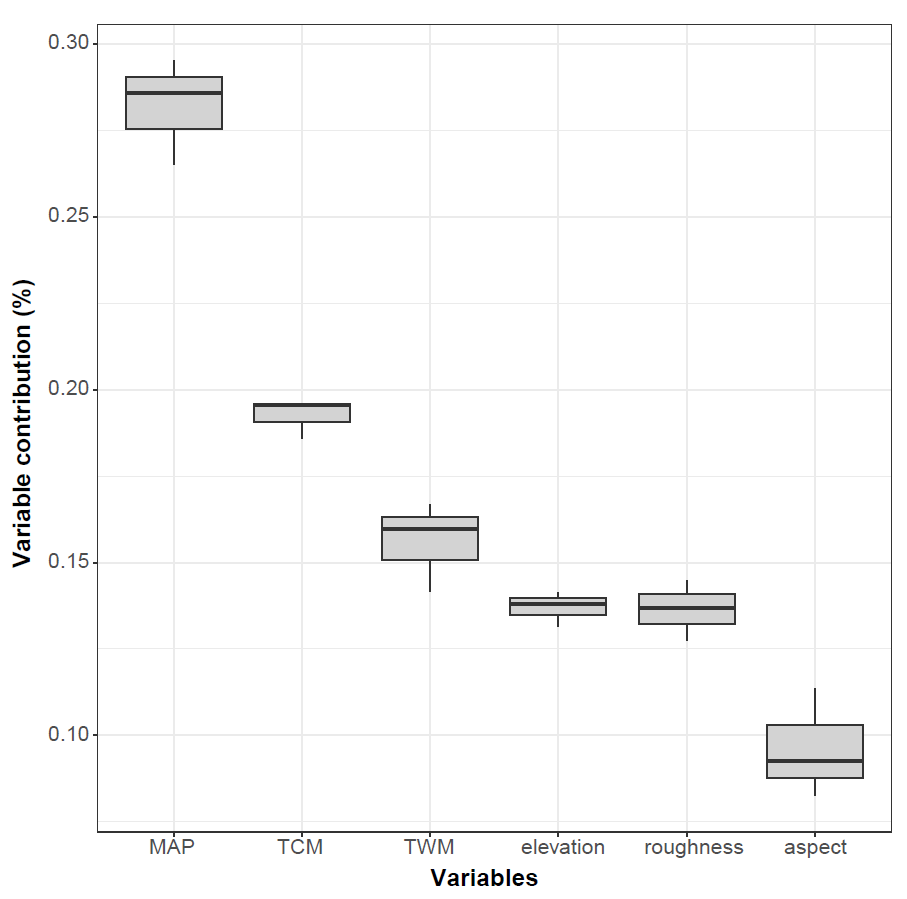


**Figure S3.** Variable contribution of non-correlated variables calculated with BART and summarized for 10 replicates for each presence-absence dataset. The variable aspect was discarded from the final models.


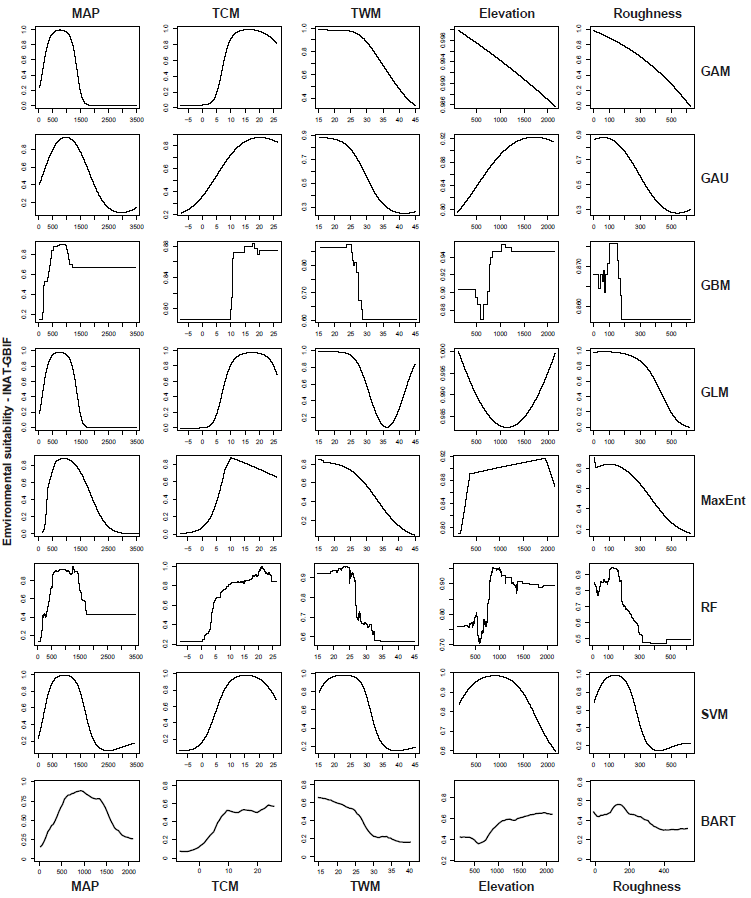


**Figure S4.** Partial dependence plots for the most important variables generated with the INAT-GBIF occurrence dataset for each of the modelling algorithms.


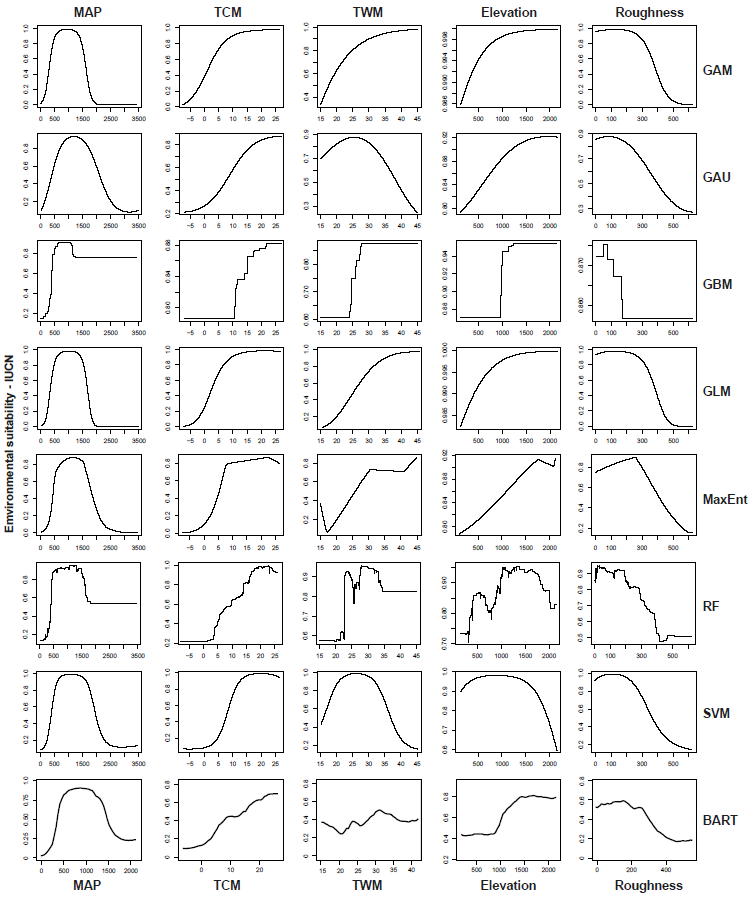


**Figure S5.** Partial dependence plots for the most important variables generated with the IUCN occurrence dataset for each of the modelling algorithms.


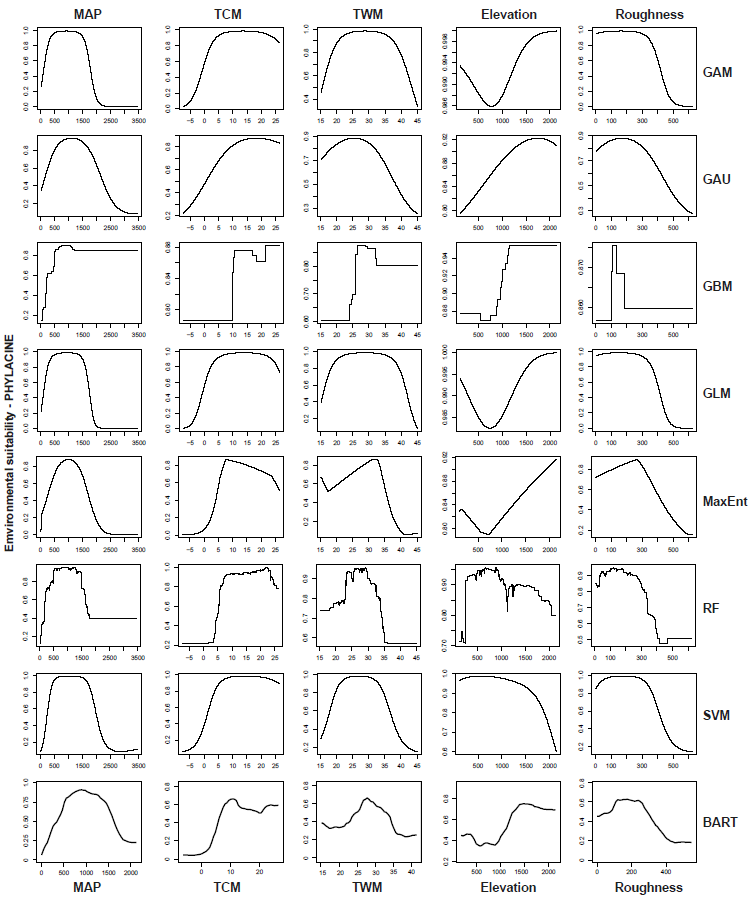


**Figure S6.** Partial dependence plots for the most important variables generated with the PHYLACINE occurrence dataset for each of the modelling algorithms.


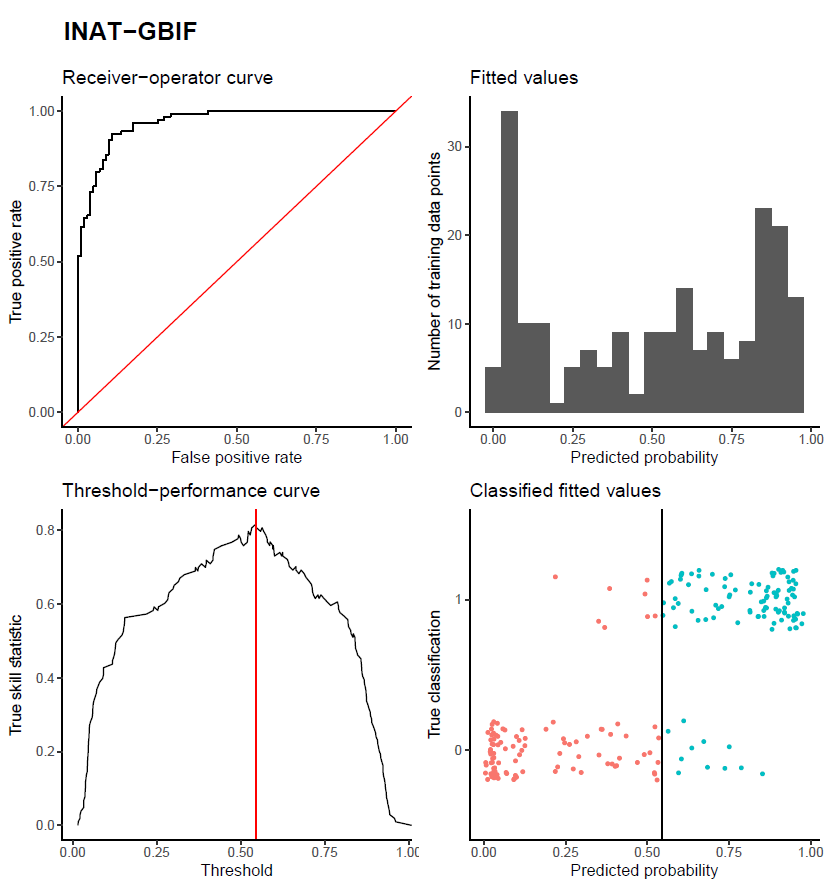


**Figure S7.** Example of the evaluation of BART of one of the 10 presence - (pseudo) absence data points generated for the INAT-GBIF occurrence dataset.


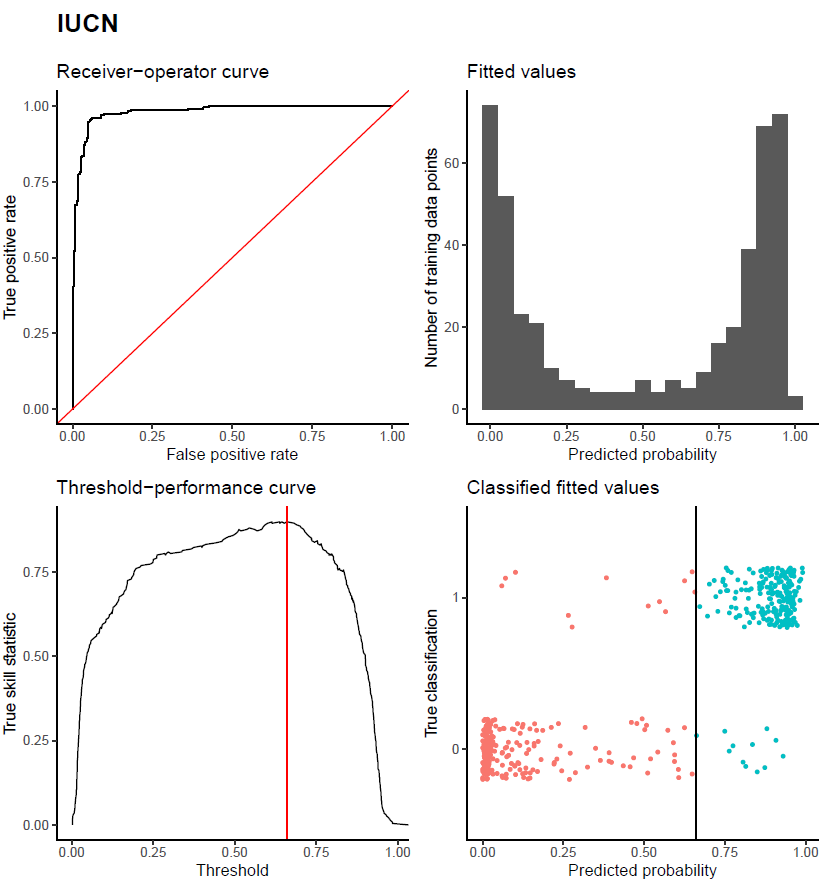


**Figure S8.** Example of the evaluation of BART of one of the 10 presence - (pseudo) absence data points generated for the IUCN occurrence dataset.


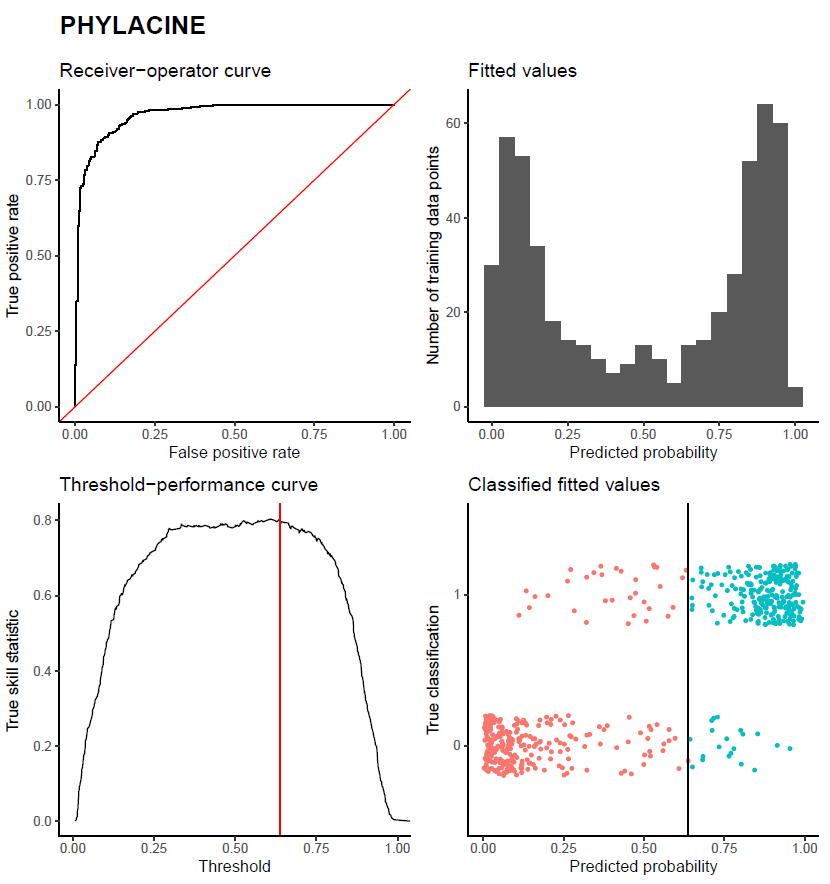


**Figure S9.** Example of the evaluation of BART of one of the 10 presence - (pseudo) absence data points generated for the PHYLACINE occurrence dataset.


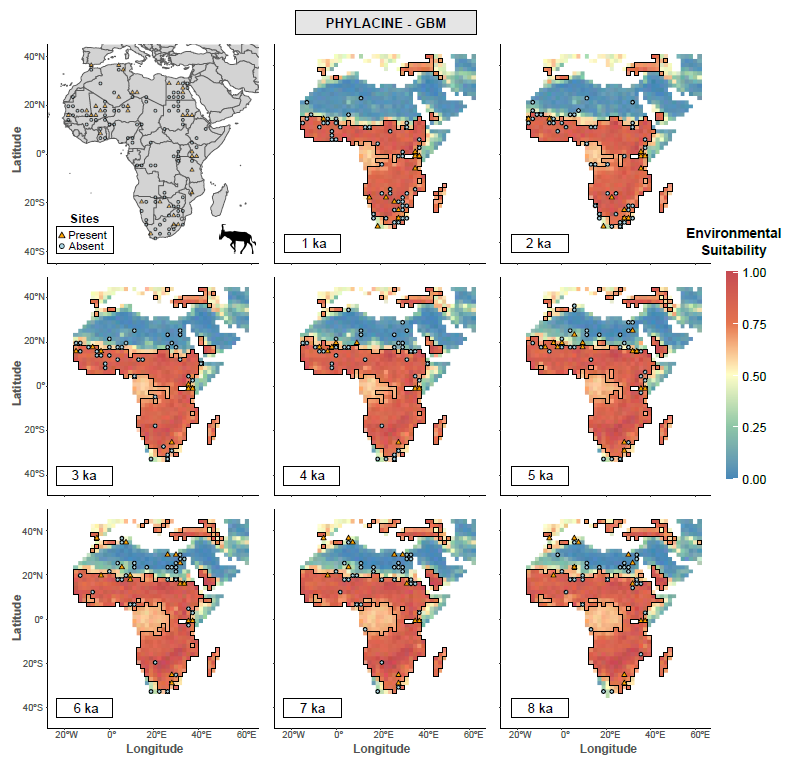


**Figure S10.** Projections of hartebeest´s environmental suitability for each of the eight 1000-yr time slices (from 8 ka to 1 ka) using the PHYLACINE occurrence dataset and the GBM model. Environmental suitability scores vary from 0 (unsuitable) to 1 (ideal conditions). The areas delimited by the black lines indicate suitable habitats above the model´s optimal threshold. The black crosses indicate the location of a site with identified hartebeest remains in that time slice.

**
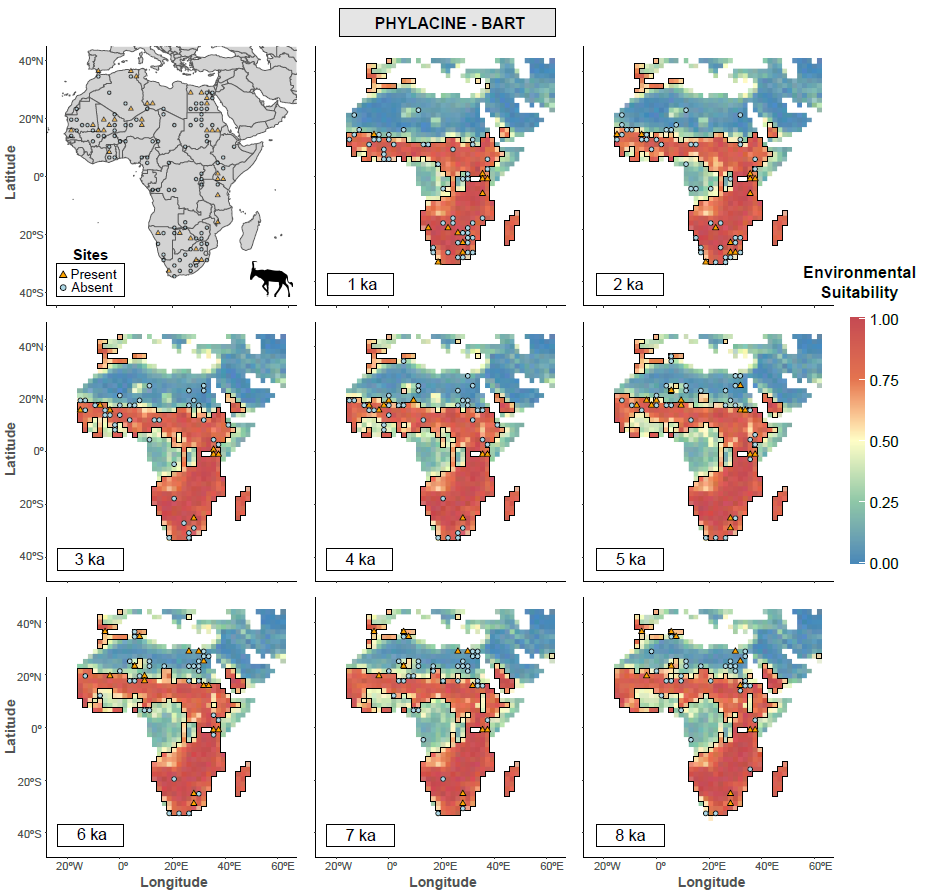
**

**Figure S11.** Projections of hartebeest´s environmental suitability for each of the eight 1000-yr time slices (from 8 ka to 1 ka) using the PHYLACINE occurrence dataset and the BART model. Environmental suitability scores vary from 0 (unsuitable) to 1 (ideal conditions). The areas delimited by the black lines indicate suitable habitats above the model´s optimal threshold. The black crosses indicate the location of a site with identified hartebeest remains in that time slice.


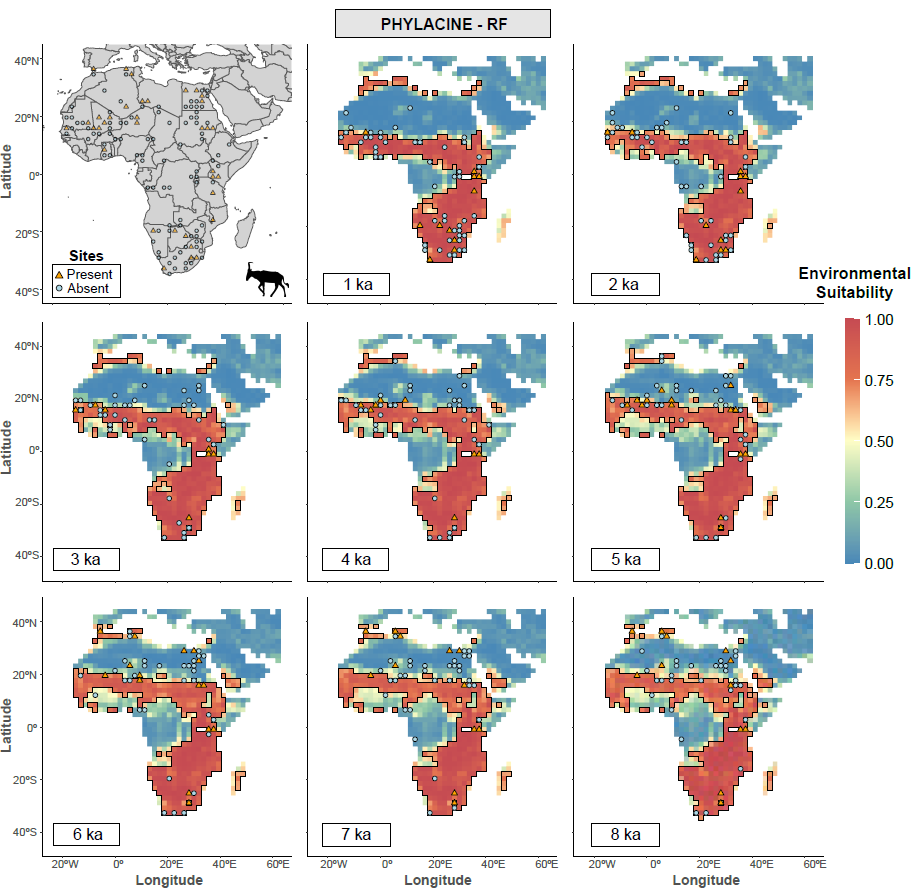


**Figure S12.** Projections of hartebeest´s environmental suitability for each of the eight 1000-yr time slices (from 8 ka to 1 ka) using the PHYLACINE occurrence dataset and the RF model. Environmental suitability scores vary from 0 (unsuitable) to 1 (ideal conditions). The areas delimited by the black lines indicate suitable habitats above the model´s optimal threshold. The black crosses indicate the location of a site with identified hartebeest remains in that time slice.


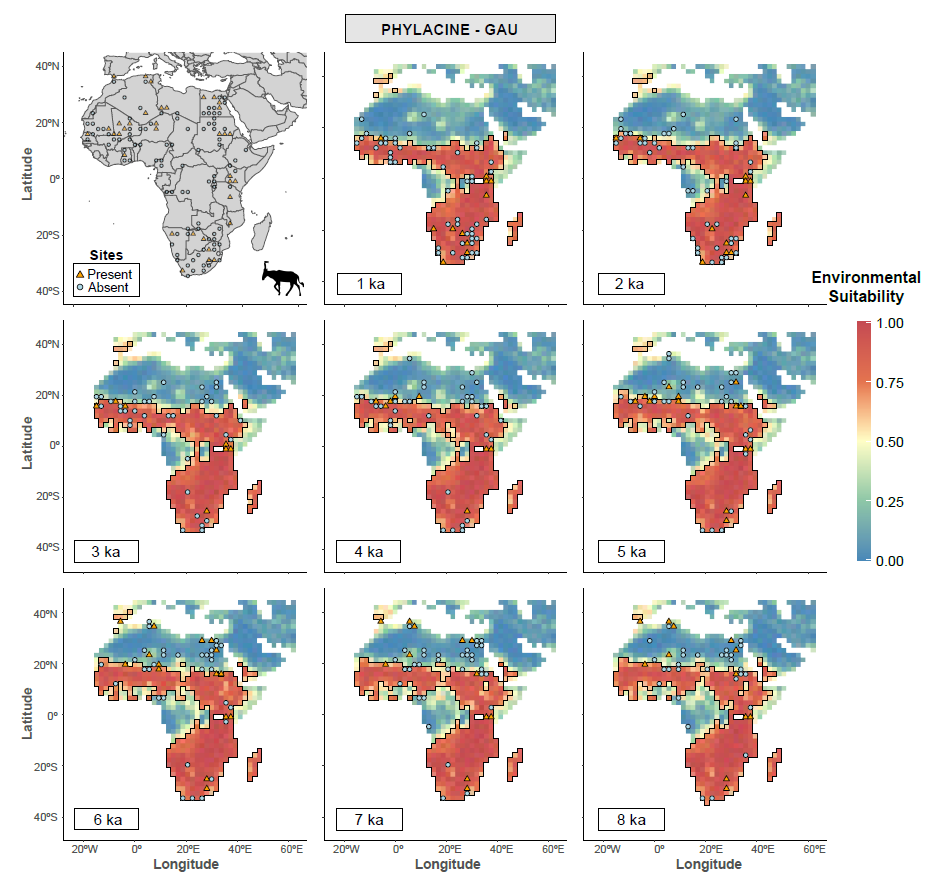


**Figure S13.** Projections of hartebeest´s environmental suitability for each of the eight 1000-yr time slices (from 8 ka to 1 ka) using the PHYLACINE occurrence dataset and the GAU model. Environmental suitability scores vary from 0 (unsuitable) to 1 (ideal conditions). The areas delimited by the black lines indicate suitable habitats above the model´s optimal threshold. The black crosses indicate the location of a site with identified hartebeest remains in that time slice.


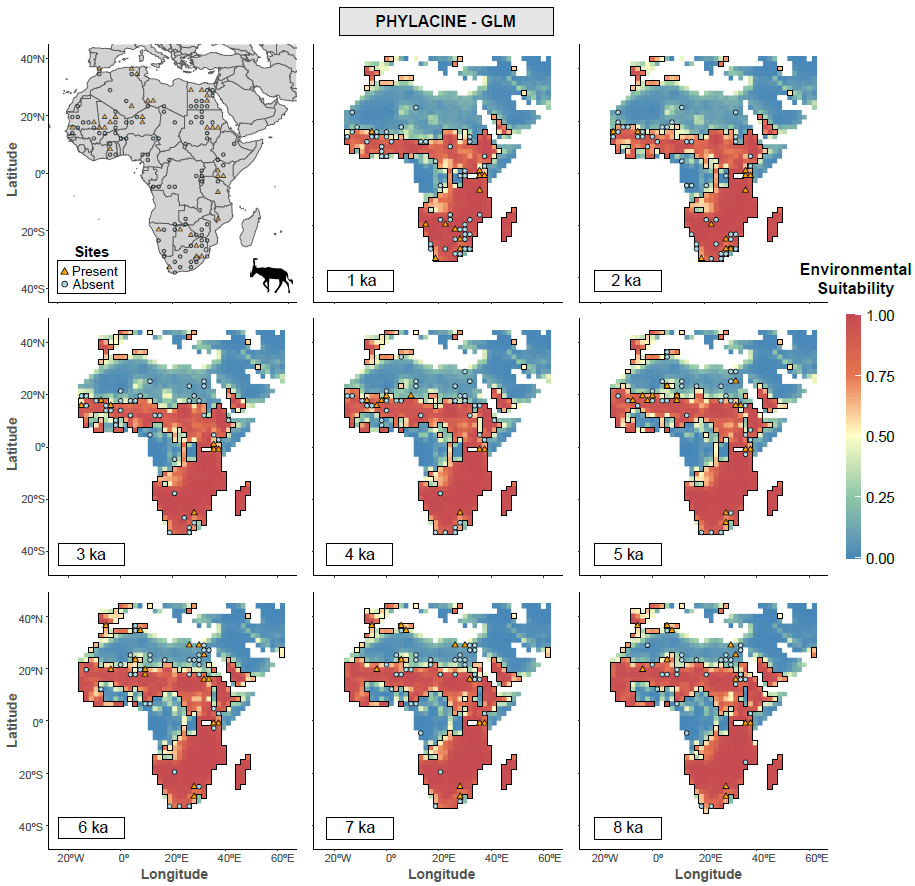


**Figure S14.** Projections of hartebeest´s environmental suitability for each of the eight 1000-yr time slices (from 8 ka to 1 ka) using the PHYLACINE occurrence dataset and the GLM model. Environmental suitability scores vary from 0 (unsuitable) to 1 (ideal conditions). The areas delimited by the black lines indicate suitable habitats above the model´s optimal threshold. The black crosses indicate the location of a site with identified hartebeest remains in that time slice.


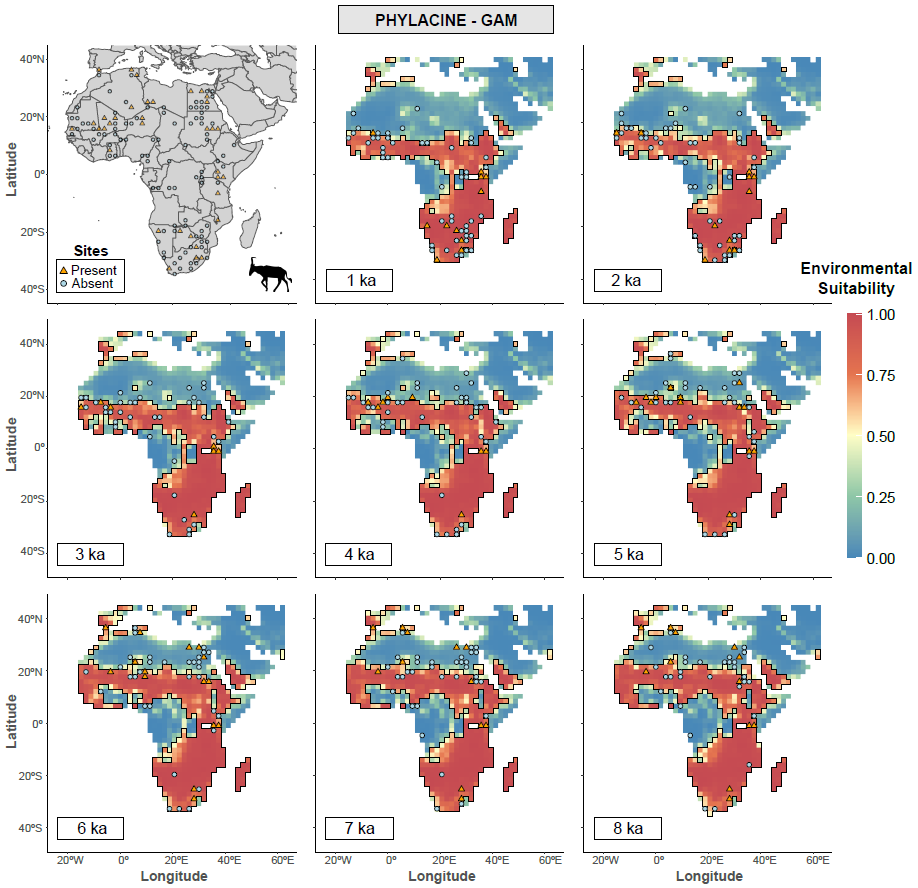


**Figure S15.** Projections of hartebeest´s environmental suitability for each of the eight 1000-yr time slices (from 8 ka to 1 ka) using the PHYLACINE occurrence dataset and the GAM model. Environmental suitability scores vary from 0 (unsuitable) to 1 (ideal conditions). The areas delimited by the black lines indicate suitable habitats above the model´s optimal threshold. The black crosses indicate the location of a site with identified hartebeest remains in that time slice.


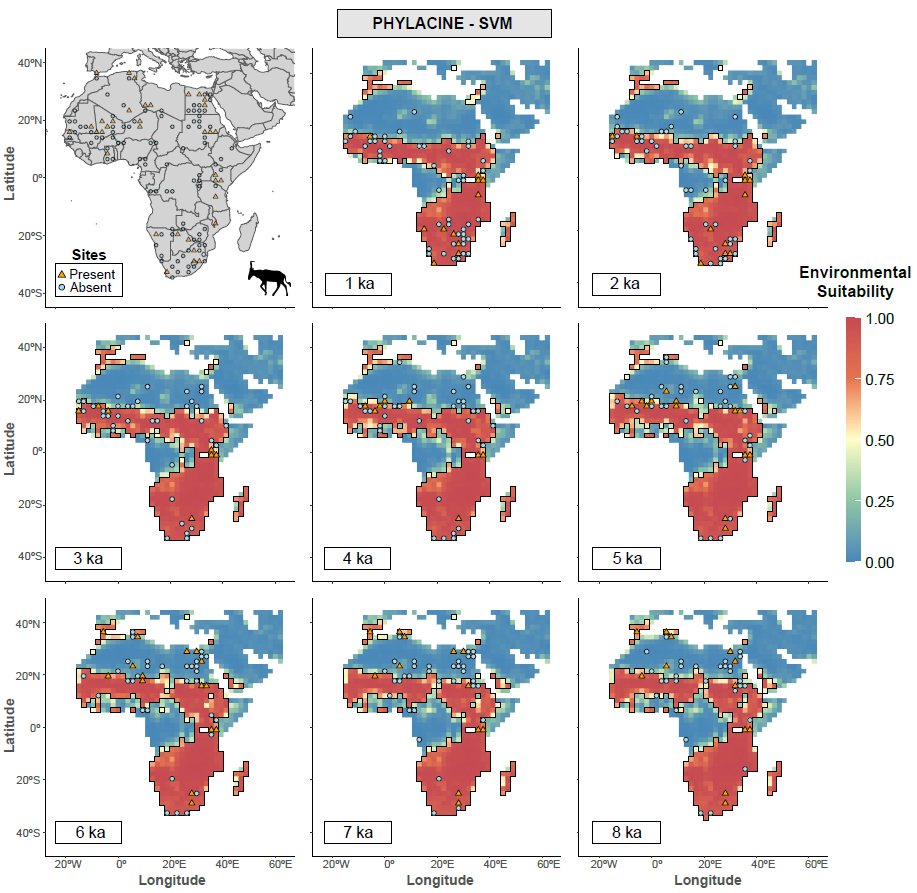


**Figure S16.** Projections of hartebeest´s environmental suitability for each of the eight 1000-yr time slices (from 8 ka to 1 ka) using the PHYLACINE occurrence dataset and the SVM model. Environmental suitability scores vary from 0 (unsuitable) to 1 (ideal conditions). The areas delimited by the black lines indicate suitable habitats above the model´s optimal threshold. The black crosses indicate the location of a site with identified hartebeest remains in that time slice.


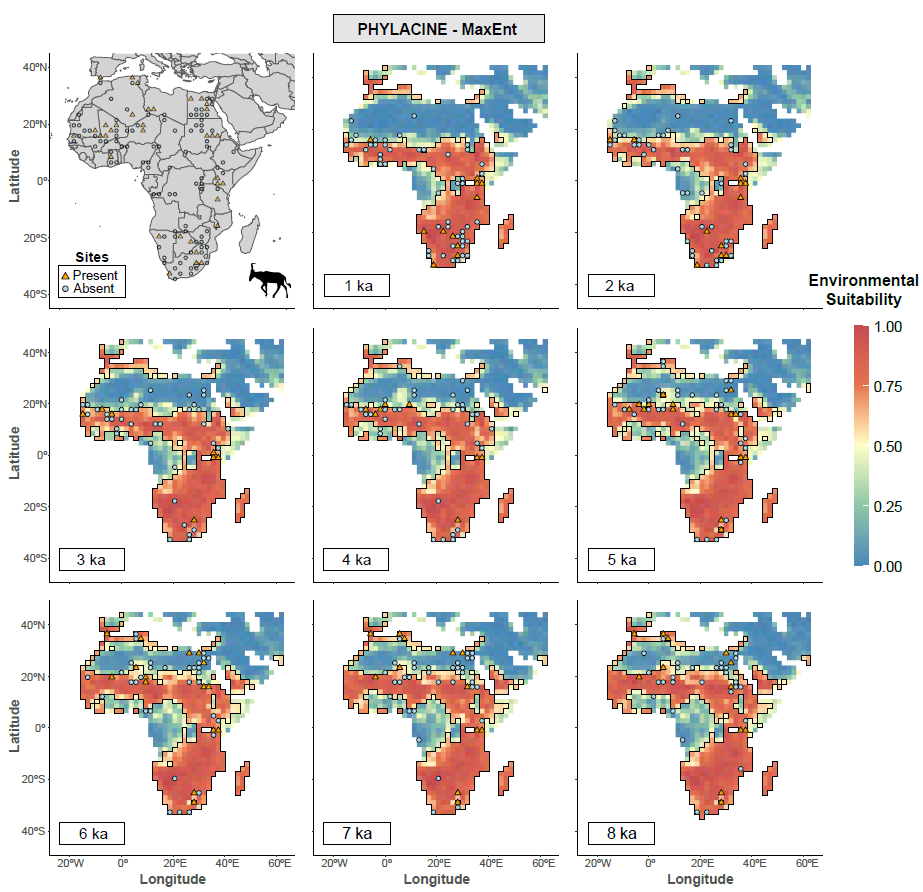


**Figure S17.** Projections of hartebeest´s environmental suitability for each of the eight 1000-yr time slices (from 8 ka to 1 ka) using the PHYLACINE occurrence dataset and the MaxEnt model. Environmental suitability scores vary from 0 (unsuitable) to 1 (ideal conditions). The areas delimited by the black lines indicate suitable habitats above the model´s optimal threshold. The black crosses indicate the location of a site with identified hartebeest remains in that time slice.


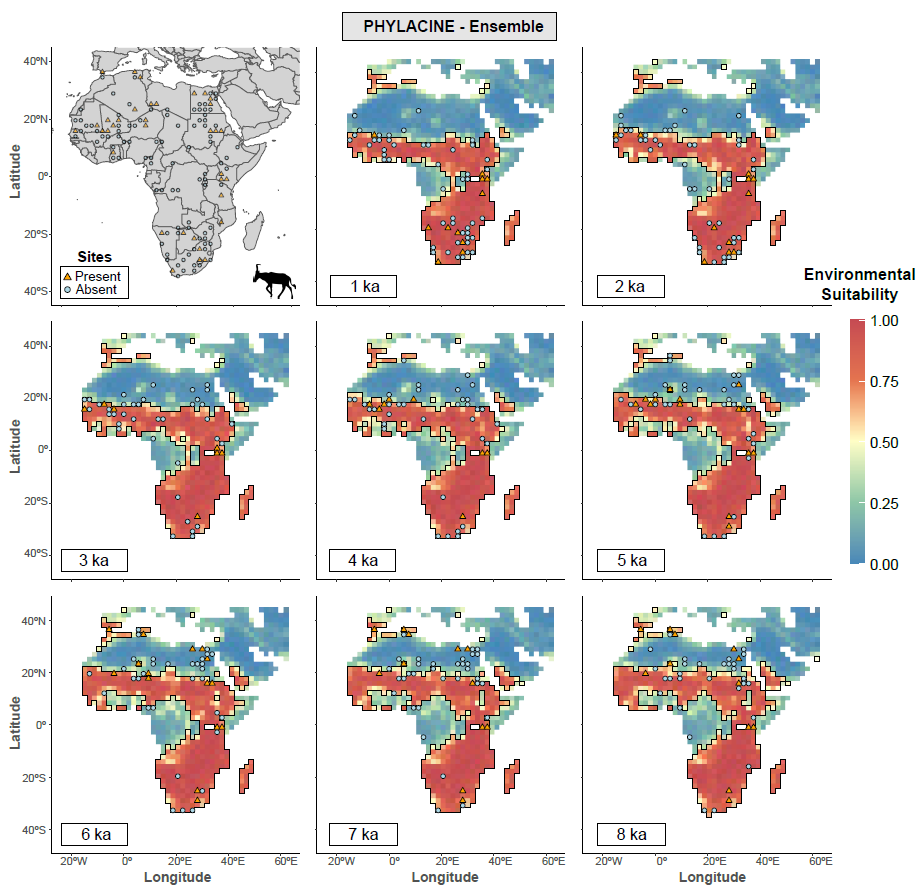


**Figure S18.** Projections of hartebeest´s environmental suitability for each of the eight 1000-yr time slices (from 8 ka to 1 ka) using the PHYLACINE occurrence dataset and the Ensemble model. Environmental suitability scores vary from 0 (unsuitable) to 1 (ideal conditions). The areas delimited by the black lines indicate suitable habitats above the model´s optimal threshold. The black crosses indicate the location of a site with identified hartebeest remains in that time slice.


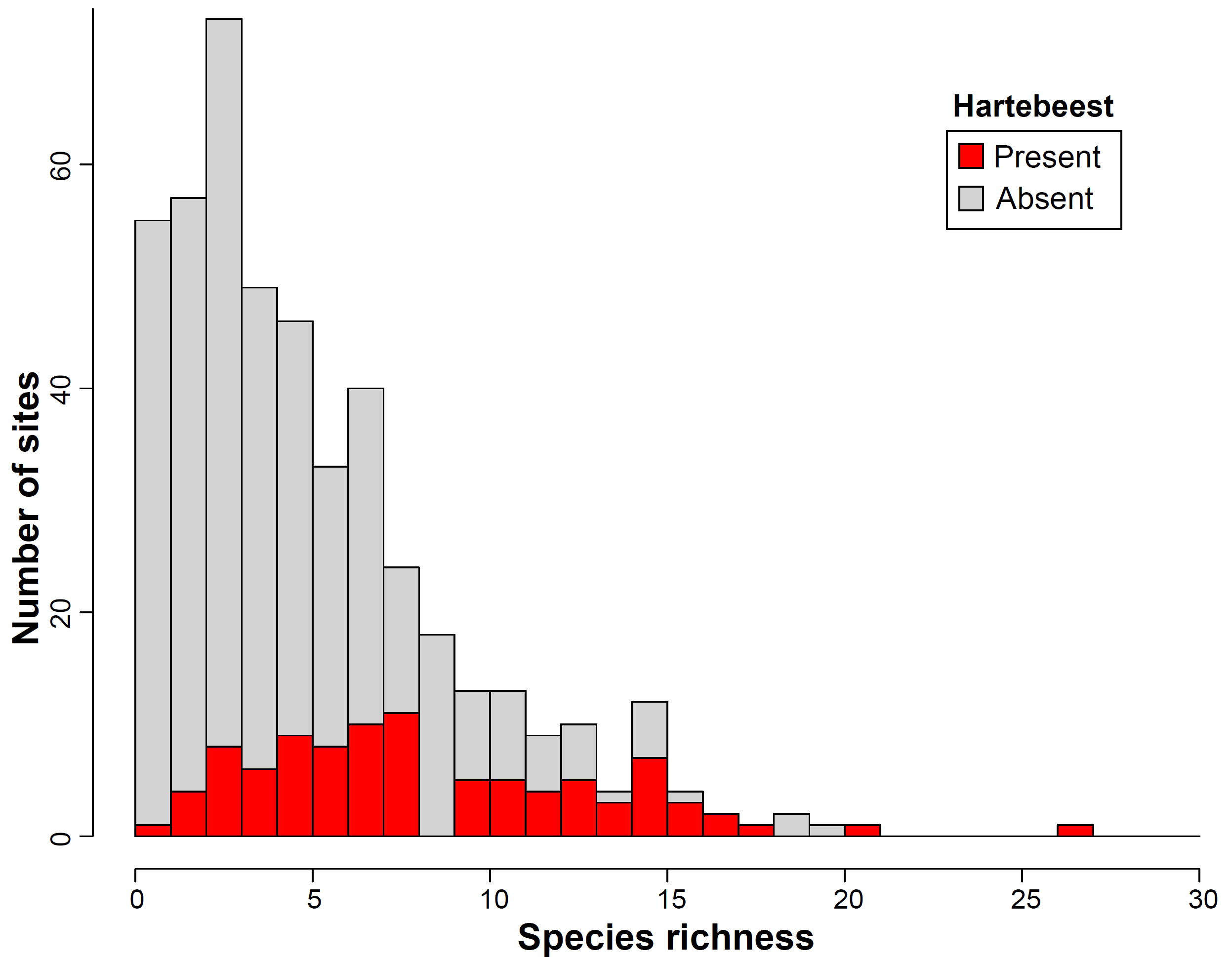


**Figure S19.** Frequency of sites with variable species richness in the paleozoological record and colored by presence (red) and absence (grey) of hartebeest.

**References**

[Acevedo, P., Jiménez-Valverde, A., Lobo, J. M., & Real, R. (2012). Delimiting the geographical background in species distribution modelling. *Journal of Biogeography*, *39*(8), 1383–1390. https://doi.org/](http://paperpile.com/b/PyavUR/kArpv)[10.1111/j.1365-2699.2012.02713.x](http://dx.doi.org/10.1111/j.1365-2699.2012.02713.x)

[Allouche, O., Tsoar, A., & Kadmon, R. (2006). Assessing the accuracy of species distribution models: prevalence, kappa and the true skill statistic (TSS). *The Journal of Applied Ecology*, *43*(6), 1223–1232. https://doi.org/](http://paperpile.com/b/PyavUR/GqNh)[10.1111/j.1365-2664.2006.01214.x](http://dx.doi.org/10.1111/j.1365-2664.2006.01214.x)

[Araújo, M. B., Anderson, R. P., Márcia Barbosa, A., Beale, C. M., Dormann, C. F., Early, R., Garcia, R. A., Guisan, A., Maiorano, L., Naimi, B., O’Hara, R. B., Zimmermann, N. E., & Rahbek, C. (2019). Standards for distribution models in biodiversity assessments. *Science Advances*, *5*(1), eaat4858. https://doi.org/](http://paperpile.com/b/PyavUR/uHksp)[10.1126/sciadv.aat4858](http://dx.doi.org/10.1126/sciadv.aat4858)

[Araújo, M. B., & Guisan, A. (2006). Five (or so) challenges for species distribution modelling. *Journal of Biogeography*, *33*(10), 1677–1688. https://doi.org/](http://paperpile.com/b/PyavUR/ZSpeR)[10.1111/j.1365-2699.2006.01584.x](http://dx.doi.org/10.1111/j.1365-2699.2006.01584.x)

[Austin, M. (2007). Species distribution models and ecological theory: A critical assessment and some possible new approaches. *Ecological Modelling*, *200*(1), 1–19. https://doi.org/](http://paperpile.com/b/PyavUR/FB1bc)[10.1016/j.ecolmodel.2006.07.005](http://dx.doi.org/10.1016/j.ecolmodel.2006.07.005)

[Austin, M. P., & Van Niel, K. P. (2011). Improving species distribution models for climate change studies: variable selection and scale. *Journal of Biogeography*, *38*(1), 1–8. https://doi.org/](http://paperpile.com/b/PyavUR/ofzDm)[10.1111/j.1365-2699.2010.02416.x](http://dx.doi.org/10.1111/j.1365-2699.2010.02416.x)

[Barbet-Massin, M., Jiguet, F., Albert, C. H., & Thuiller, W. (2012). Selecting pseudo-absences for species distribution models: how, where and how many? *Methods in Ecology and Evolution / British Ecological Society*, *3*(2), 327–338. https://doi.org/](http://paperpile.com/b/PyavUR/J6iQf)[10.1111/j.2041-210x.2011.00172.x](http://dx.doi.org/10.1111/j.2041-210x.2011.00172.x)

[Barbet-Massin, M., Thuiller, W., & Jiguet, F. (2010). How much do we overestimate future local extinction rates when restricting the range of occurrence data in climate suitability models? *Ecography*, *33*(5), 878–886. https://doi.org/](http://paperpile.com/b/PyavUR/dUtHg)[10.1111/j.1600-0587.2010.06181.x](http://dx.doi.org/10.1111/j.1600-0587.2010.06181.x)

[Barbosa, A. M. (2015). fuzzySim: applying fuzzy logic to binary similarity indices in ecology.](http://paperpile.com/b/PyavUR/N2CB) *Methods in Ecology and Evolution*, [*6*](file:///G:\My%20Drive\MAIN\RESEARCH%20PROJECTS\MS%20IN%20PREP\2024_Lazagabaster.et.al_EcolEvolution_Hartebeest_SDMs\EE%20submission_reviewed\ms\6)[(7), 853–858.](file:///G:\My%20Drive\MAIN\RESEARCH%20PROJECTS\MS%20IN%20PREP\2024_Lazagabaster.et.al_EcolEvolution_Hartebeest_SDMs\EE%20submission_reviewed\ms\(7),%20853–858) [https://doi.org/](%20https://doi.org/)[10.1111/2041-210X.12372](http://dx.doi.org/10.1111/2041-210X.12372)

[Barbosa, A. M., Brown, J., Jiménez-Valverde, A., & Real, R. (2016). *ModEvA: Model evaluation and analysis*.](http://paperpile.com/b/PyavUR/hZnf) <https://dspace.uevora.pt/rdpc/handle/10174/20946>

[Barbosa, A. M., Real, R., & Vargas, J. M. (2010). Use of coarse-resolution models of species’ distributions to guide local conservation inferences. *Conservation Biology: The Journal of the Society for Conservation Biology*, *24*(5), 1378–1387. https://doi.org/](http://paperpile.com/b/PyavUR/yCZkk)[10.1111/j.1523-1739.2010.01517.x](http://dx.doi.org/10.1111/j.1523-1739.2010.01517.x)

[Barve, V., Hart, E., & Guillou, S. (2022). rinat: Access’ iNaturalist' data through APIs. *R Package Version 0.1*.](http://paperpile.com/b/PyavUR/F9Iw) https://docs.ropensci.org/rinat/

[Beck, J., Böller, M., Erhardt, A., & Schwanghart, W. (2014). Spatial bias in the GBIF database and its effect on modeling species’ geographic distributions. *Ecological Informatics*, *19*, 10–15. https://doi.org/](http://paperpile.com/b/PyavUR/cXfsH)[10.1016/j.ecoinf.2013.11.002](http://dx.doi.org/10.1016/j.ecoinf.2013.11.002)

[Beever, E. A., Hall, L. E., Varner, J., Loosen, A. E., Dunham, J. B., Gahl, M. K., Smith, F. A., & Lawler, J. J. (2017). Behavioral flexibility as a mechanism for coping with climate change. *Frontiers in Ecology and the Environment*, *15*(6), 299–308. https://doi.org/](http://paperpile.com/b/PyavUR/Qh6V8)[10.1002/fee.1502](http://dx.doi.org/10.1002/fee.1502)

[Carl, G., & Kühn, I. (2007). Analyzing spatial autocorrelation in species distributions using Gaussian and logit models. *Ecological Modelling*, *207*(2), 159–170. https://doi.org/](http://paperpile.com/b/PyavUR/4HbI0)[10.1016/j.ecolmodel.2007.04.024](http://dx.doi.org/10.1016/j.ecolmodel.2007.04.024)

[Carlson, C. J. (2020). embarcadero: Species distribution modelling with Bayesian additive regression trees in r. *Methods in Ecology and Evolution / British Ecological Society*, *11*(7), 850–858. https://doi.org/](http://paperpile.com/b/PyavUR/Y0Gu)[10.1111/2041-210x.13389](http://dx.doi.org/10.1111/2041-210x.13389)

[Chamaillé‐Jammes, S., & Fritz, H. (2009). Precipitation–NDVI relationships in eastern and southern African savannas vary along a precipitation gradient. *International Journal of Remote Sensing*, *30*(13), 3409–3422. https://doi.org/](http://paperpile.com/b/PyavUR/H5x9A)[10.1080/01431160802562206](http://dx.doi.org/10.1080/01431160802562206)

[Chamberlain, S., Oldoni, D., Geffert, L., Desmet, P., Barve, V., Ram, K., Blissett, M., Waller, J., McGlinn, D., Ooms, J., Ye, S. (siwei), Oksanen, J., Marwick, B., John, Sumner, M., & Sriram. (2022). *ropensci/rgbif: rgbif v3.7.0*. Zenodo. https://doi.org/](http://paperpile.com/b/PyavUR/qCA6)[10.5281/ZENODO.6023735](http://dx.doi.org/10.5281/ZENODO.6023735)

[Chapman, D., Pescott, O. L., Roy, H. E., & Tanner, R. (2019). Improving species distribution models for invasive non‐native species with biologically informed pseudo‐absence selection. *Journal of Biogeography*, *46*(5), 1029–1040. https://doi.org/](http://paperpile.com/b/PyavUR/JBQuK)[10.1111/jbi.13555](http://dx.doi.org/10.1111/jbi.13555)

[Chipman, H. A., George, E. I., & McCulloch, R. E. (2010). BART: Bayesian additive regression trees. *The Annals of Applied Statistics*, *4*(1), 266–298. https://doi.org/](http://paperpile.com/b/PyavUR/kshmQ)[10.1214/09-AOAS285](http://dx.doi.org/10.1214/09-AOAS285)

[Cobos, M. E., Peterson, A. T., Osorio-Olvera, L., & Jiménez-García, D. (2019). An exhaustive analysis of heuristic methods for variable selection in ecological niche modeling and species distribution modeling. *Ecological Informatics*, *53*, 100983. https://doi.org/](http://paperpile.com/b/PyavUR/mhFDH)[10.1016/j.ecoinf.2019.100983](http://dx.doi.org/10.1016/j.ecoinf.2019.100983)

[Craney, T. A., & Surles, J. G. (2002). Model-dependent variance inflation factor cutoff values. *Quality Engineering*, *14*(3), 391–403. https://doi.org/](http://paperpile.com/b/PyavUR/zNcuQ)[10.1081/qen-120001878](http://dx.doi.org/10.1081/qen-120001878)

[Cutler, D. R., Edwards, T. C., Jr, Beard, K. H., Cutler, A., Hess, K. T., Gibson, J., & Lawler, J. J. (2007). Random forests for classification in ecology. *Ecology*, *88*(11), 2783–2792. https://doi.org/](http://paperpile.com/b/PyavUR/2tP6W)[10.1890/07-0539.1](http://dx.doi.org/10.1890/07-0539.1)

[Dallmeyer, A., Claussen, M., Lorenz, S. J., & Shanahan, T. (2020). The end of the African humid period as seen by a transient comprehensive Earth system model simulation of the last 8000 years. *Climate of the Past*, *16*(1), 117–140. https://doi.org/](http://paperpile.com/b/PyavUR/hZHeL)[10.5194/cp-16-117-2020](http://dx.doi.org/10.5194/cp-16-117-2020)

[Dormann, C. F., McPherson, J. M., Araújo, M. B., Bivand, R., Bolliger, J., Carl, G., Davies, R. G., Hirzel, A., Jetz, W., Kissling, W. D., Kühn, I., Ohlemüller, R., Peres-Neto, P. R., Reineking, B., Schröder, B., Schurr, F. M., & Wilson, R. (2007). Methods to Account for Spatial Autocorrelation in the Analysis of Species Distributional Data: A Review. *Ecography*, *30*(5), 609–628.](http://paperpile.com/b/PyavUR/6xXJn) <http://www.jstor.org/stable/30244511>

[Drake, J. M., Randin, C., & Guisan, A. (2006). Modelling ecological niches with support vector machines. *The Journal of Applied Ecology*, *43*(3), 424–432. https://doi.org/](http://paperpile.com/b/PyavUR/RLQ8Z)[10.1111/j.1365-2664.2006.01141.x](http://dx.doi.org/10.1111/j.1365-2664.2006.01141.x)

[Elith, J., & Leathwick, J. R. (2009). Species distribution models: Ecological explanation and prediction across space and time. *Annual Review of Ecology, Evolution, and Systematics*, *40*(1), 677–697. https://doi.org/](http://paperpile.com/b/PyavUR/RADxW)[10.1146/annurev.ecolsys.110308.120159](http://dx.doi.org/10.1146/annurev.ecolsys.110308.120159)

[Elith, J., Leathwick, J. R., & Hastie, T. (2008). A working guide to boosted regression trees. *The Journal of Animal Ecology*, *77*(4), 802–813. https://doi.org/](http://paperpile.com/b/PyavUR/NK2VS)[10.1111/j.1365-2656.2008.01390.x](http://dx.doi.org/10.1111/j.1365-2656.2008.01390.x)

[Faurby, S., Davis, M., Pedersen, R. Ø., Schowanek, S. D., Antonelli, A., & Svenning, J.-C. (2018). PHYLACINE 1.2: The Phylogenetic Atlas of Mammal Macroecology. *Ecology*, *99*(11), 2626. https://doi.org/](http://paperpile.com/b/PyavUR/6UJuP)[10.1002/ecy.2443](http://dx.doi.org/10.1002/ecy.2443)

[F. Dormann, C., M. McPherson, J., B. Araújo, M., Bivand, R., Bolliger, J., Carl, G., G. Davies, R., Hirzel, A., Jetz, W., Daniel Kissling, W., Kühn, I., Ohlemüller, R., R. Peres-Neto, P., Reineking, B., Schröder, B., M. Schurr, F., & Wilson, R. (2007). Methods to account for spatial autocorrelation in the analysis of species distributional data: a review. *Ecography*, *30*(5), 609–628. https://doi.org/](http://paperpile.com/b/PyavUR/Gv3Xu)[10.1111/j.2007.0906-7590.05171.x](http://dx.doi.org/10.1111/j.2007.0906-7590.05171.x)

[Fei, S., & Yu, F. (2016). Quality of presence data determines species distribution model performance: a novel index to evaluate data quality. *Landscape Ecology*, *31*(1), 31–42. https://doi.org/](http://paperpile.com/b/PyavUR/3fLV0)[10.1007/s10980-015-0272-7](http://dx.doi.org/10.1007/s10980-015-0272-7)

[Fourcade, Y. (2016). Comparing species distributions modelled from occurrence data and from expert-based range maps. Implication for predicting range shifts with climate change. *Ecological Informatics*, *36*, 8–14. https://doi.org/](http://paperpile.com/b/PyavUR/jGBWA)[10.1016/j.ecoinf.2016.09.002](http://dx.doi.org/10.1016/j.ecoinf.2016.09.002)

[Franklin, J. (2010). Implementation of species distribution models. In *Mapping Species Distributions* (pp. 235–261). Cambridge University Press. https://doi.org/](http://paperpile.com/b/PyavUR/pd81o)[10.1017/cbo9780511810602.015](http://dx.doi.org/10.1017/cbo9780511810602.015)

[Gastón, A., & García-Viñas, J. I. (2011). Modelling species distributions with penalised logistic regressions: A comparison with maximum entropy models. *Ecological Modelling*, *222*(13), 2037–2041. https://doi.org/](http://paperpile.com/b/PyavUR/xHc2z)[10.1016/j.ecolmodel.2011.04.015](http://dx.doi.org/10.1016/j.ecolmodel.2011.04.015)

[Golding, N., & Purse, B. V. (2016). Fast and flexible Bayesian species distribution modelling using Gaussian processes.](http://paperpile.com/b/PyavUR/ZOHhn) <https://doi.org/>[10.1111/2041-210X.12523](http://dx.doi.org/10.1111/2041-210X.12523)

[Gomes, V. H. F., IJff, S. D., Raes, N., Amaral, I. L., Salomão, R. P., de Souza Coelho, L., de Almeida Matos, F. D., Castilho, C. V., de Andrade Lima Filho, D., López, D. C., Guevara, J. E., Magnusson, W. E., Phillips, O. L., Wittmann, F., de Jesus Veiga Carim, M., Martins, M. P., Irume, M. V., Sabatier, D., Molino, J.-F., … Ter Steege, H. (2018). Species Distribution Modelling: Contrasting presence-only models with plot abundance data. *Scientific Reports*, *8*(1), 1003. https://doi.org/](http://paperpile.com/b/PyavUR/sc6w7)[10.1038/s41598-017-18927-1](http://dx.doi.org/10.1038/s41598-017-18927-1)

[Gosling LM Capellini I. (2013). Alcelaphus buselaphus Hartebeest. In Kingdon JS Hoffmann M (Ed.), *The Mammals of Africa* (pp. 511–526). Bloomsbury Publishing.](http://paperpile.com/b/PyavUR/N7sgt)

[Guisan, A., & Thuiller, W. (2005). Predicting species distribution: offering more than simple habitat models. *Ecology Letters*, *8*(9), 993–1009. https://doi.org/](http://paperpile.com/b/PyavUR/DgiPD)[10.1111/j.1461-0248.2005.00792.x](http://dx.doi.org/10.1111/j.1461-0248.2005.00792.x)

[Guisan, A., Thuiller, W., & Zimmermann, N. E. (2017). Environmental predictors: Issues of processing and selection. In *Habitat Suitability and Distribution Models* (pp. 61–109). Cambridge University Press. https://doi.org/](http://paperpile.com/b/PyavUR/JjESI)[10.1017/9781139028271.011](http://dx.doi.org/10.1017/9781139028271.011)

[Guo, Q., Kelly, M., & Graham, C. H. (2005). Support vector machines for predicting distribution of Sudden Oak Death in California. *Ecological Modelling*, *182*(1), 75–90. https://doi.org/](http://paperpile.com/b/PyavUR/3EuSq)[10.1016/j.ecolmodel.2004.07.012](http://dx.doi.org/10.1016/j.ecolmodel.2004.07.012)

[Hallgren, W., Santana, F., Low-Choy, S., Zhao, Y., & Mackey, B. (2019). Species distribution models can be highly sensitive to algorithm configuration. *Ecological Modelling*, *408*, 108719. https://doi.org/](http://paperpile.com/b/PyavUR/mY2HZ)[10.1016/j.ecolmodel.2019.108719](http://dx.doi.org/10.1016/j.ecolmodel.2019.108719)

[Harris, R. M. B., Porfirio, L. L., Hugh, S., Lee, G., Bindoff, N. L., Mackey, B., & Beeton, N. J. (2013). To Be Or Not to Be? Variable selection can change the projected fate of a threatened species under future climate. *Ecological Management & Restoration*. https://doi.org/](http://paperpile.com/b/PyavUR/2QxtU)[10.1111/emr.12055](http://dx.doi.org/10.1111/emr.12055)

[Hijmans, R. J., Bivand, R., Forner, K., Ooms, J., Pebesma, E., & Sumner, M. D. (2022). Package “terra.” *Maintainer: Vienna, Austria*.](http://paperpile.com/b/PyavUR/jCPp)

[Hopcroft, P. O., & Valdes, P. J. (2022). Green Sahara tipping points in transient climate model simulations of the Holocene. *Environmental Research Letters: ERL [Web Site]*, *17*(8), 085001. https://doi.org/](http://paperpile.com/b/PyavUR/KsDRz)[10.1088/1748-9326/ac7c2b](http://dx.doi.org/10.1088/1748-9326/ac7c2b)

[Iturbide, M., Bedia, J., Herrera, S., del Hierro, O., Pinto, M., & Gutiérrez, J. M. (2015). A framework for species distribution modelling with improved pseudo-absence generation. *Ecological Modelling*, *312*, 166–174. https://doi.org/](http://paperpile.com/b/PyavUR/hfA7u)[10.1016/j.ecolmodel.2015.05.018](http://dx.doi.org/10.1016/j.ecolmodel.2015.05.018)

[Janizadeh, S., Vafakhah, M., Kapelan, Z., & Dinan, N. M. (2021). Novel Bayesian Additive Regression Tree Methodology for Flood Susceptibility Modeling. *Water Resources Management*, *35*(13), 4621–4646. https://doi.org/](http://paperpile.com/b/PyavUR/SuPw8)[10.1007/s11269-021-02972-7](http://dx.doi.org/10.1007/s11269-021-02972-7)

[Karger, Nobis, & Normand. (2021). CHELSA-TraCE21k v1. 0. Downscaled transient temperature and precipitation data since the last glacial maximum. *Climate of the Past*.](http://paperpile.com/b/PyavUR/gxQCz) <https://cp.copernicus.org/preprints/cp-2021-30/>

[Kass, J. M., Meenan, S. I., Tinoco, N., Burneo, S. F., & Anderson, R. P. (2021). Improving area of occupancy estimates for parapatric species using distribution models and support vector machines. *Ecological Applications: A Publication of the Ecological Society of America*, *31*(1), e02228. https://doi.org/](http://paperpile.com/b/PyavUR/XX4te)[10.1002/eap.2228](http://dx.doi.org/10.1002/eap.2228)

[Kingdon, J. (2015). *The Kingdon Field Guide to African Mammals: Second Edition*](http://paperpile.com/b/PyavUR/x6jqJ)

[Lee, W.-H., Song, J.-W., Yoon, S.-H., & Jung, J.-M. (2022). Spatial Evaluation of Machine Learning-Based Species Distribution Models for Prediction of Invasive Ant Species Distribution. *NATO Advanced Science Institutes Series E: Applied Sciences*, *12*(20), 10260. https://doi.org/](http://paperpile.com/b/PyavUR/p3nAk)[10.3390/app122010260](http://dx.doi.org/10.3390/app122010260)

[Lee-Yaw, J. A., McCune, J. L., Pironon, S., & Sheth, S. N. (2022). Species distribution models rarely predict the biology of real populations. *Ecography*, *2022*(6). https://doi.org/](http://paperpile.com/b/PyavUR/FexSz)[10.1111/ecog.05877](http://dx.doi.org/10.1111/ecog.05877)

[Lichstein, J. W., Simons, T. R., & Shriner, S. A. (2002). Spatial autocorrelation and autoregressive models in ecology. *Ecological*](http://paperpile.com/b/PyavUR/Se7NE) *monographs*[. https://doi.org/](file:///G:\My%20Drive\MAIN\RESEARCH%20PROJECTS\MS%20IN%20PREP\2024_Lazagabaster.et.al_EcolEvolution_Hartebeest_SDMs\EE%20submission_reviewed\ms\.%20https:\doi.org\)[10.1890/0012-9615(2002)072[0445:SAAAMI]2.0.CO;2](http://dx.doi.org/10.1890/0012-9615(2002)072%5B0445:SAAAMI%5D2.0.CO;2)

[Liu, Z. (2018). *Bayesian Classification Methods for Bat Call Identification* [Washington University in St. Louis]. https://doi.org/](http://paperpile.com/b/PyavUR/VRVg0)[10.7936/K7V1247W](http://dx.doi.org/10.7936/K7V1247W)

[Li, X., & Wang, Y. (2013). Applying various algorithms for species distribution modelling. *Integrative Zoology*, *8*(2), 124–135. https://doi.org/](http://paperpile.com/b/PyavUR/fs6n4)[10.1111/1749-4877.12000](http://dx.doi.org/10.1111/1749-4877.12000)

[Lobo, J. M., Jiménez-Valverde, A., & Hortal, J. (2010). The uncertain nature of absences and their importance in species distribution modelling. *Ecography*, *33*(1), 103–114. https://doi.org/](http://paperpile.com/b/PyavUR/3gnHe)[10.1111/j.1600-0587.2009.06039.x](http://dx.doi.org/10.1111/j.1600-0587.2009.06039.x)

[Marmion, M., Parviainen, M., Luoto, M., Heikkinen, R. K., & Thuiller, W. (2009). Evaluation of consensus methods in predictive species distribution modelling. *Diversity & Distributions*, *15*(1), 59–69. https://doi.org/](http://paperpile.com/b/PyavUR/4r06B)[10.1111/j.1472-4642.2008.00491.x](http://dx.doi.org/10.1111/j.1472-4642.2008.00491.x)

[Martiny, N., Camberlin, P., Richard, Y., & Philippon, N. (2006). Compared regimes of NDVI and rainfall in semi‐arid regions of Africa. *International Journal of Remote Sensing*, *27*(23), 5201–5223. https://doi.org/](http://paperpile.com/b/PyavUR/cQLjQ)[10.1080/01431160600567787](http://dx.doi.org/10.1080/01431160600567787)

[McCord, S. E., Buenemann, M., Karl, J. W., Browning, D. M., & Hadley, B. C. (2017). Integrating Remotely Sensed Imagery and Existing Multiscale Field Data to Derive Rangeland Indicators: Application of Bayesian Additive Regression Trees. *Rangeland Ecology & Management*, *70*(5), 644–655. https://doi.org/](http://paperpile.com/b/PyavUR/ZWApn)[10.1016/j.rama.2017.02.004](http://dx.doi.org/10.1016/j.rama.2017.02.004)

[Melo-Merino, S. M., Reyes-Bonilla, H., & Lira-Noriega, A. (2020). Ecological niche models and species distribution models in marine environments: A literature review and spatial analysis of evidence. *Ecological Modelling*, *415*, 108837. https://doi.org/](http://paperpile.com/b/PyavUR/fKxAZ)[10.1016/j.ecolmodel.2019.108837](http://dx.doi.org/10.1016/j.ecolmodel.2019.108837)

[Merow, C., Allen, J. M., Aiello-Lammens, M., Silander, J. A., Jr, & Fortin, M.-J. (2016). Improving niche and range estimates with Maxent and point process models by integrating spatially explicit information. *Global Ecology and Biogeography: A Journal of Macroecology*, *25*(8), 1022–1036. https://doi.org/](http://paperpile.com/b/PyavUR/Oj1ks)[10.1111/geb.12453](http://dx.doi.org/10.1111/geb.12453)

[Pearce, J., & Ferrier, S. (2000). An evaluation of alternative algorithms for fitting species distribution models using logistic regression. *Ecological Modelling*, *128*(2), 127–147. https://doi.org/](http://paperpile.com/b/PyavUR/qqviW)[10.1016/S0304-3800(99)00227-6](http://dx.doi.org/10.1016/S0304-3800(99)00227-6)

[Petitpierre, B., Broennimann, O., Kueffer, C., Daehler, C., & Guisan, A. (2017). Selecting predictors to maximize the transferability of species distribution models: lessons from cross-continental plant invasions. *Global Ecology and Biogeography: A Journal of Macroecology*, *26*(3), 275–287. https://doi.org/](http://paperpile.com/b/PyavUR/6TacD)[10.1111/geb.12530](http://dx.doi.org/10.1111/geb.12530)

[Phillips, S. J., Dudík, M., Elith, J., Graham, C. H., Lehmann, A., Leathwick, J., & Ferrier, S. (2009). Sample selection bias and presence-only distribution models: implications for background and pseudo-absence data. *Ecological Applications: A Publication of the Ecological Society of America*, *19*(1), 181–197. https://doi.org/](http://paperpile.com/b/PyavUR/OQKSh)[10.1890/07-2153.1](http://dx.doi.org/10.1890/07-2153.1)

[Plant, E., King, R., & Kath, J. (2021). Statistical comparison of additive regression tree methods on ecological grassland data. *Ecological Informatics*, *61*, 101198. https://doi.org/](http://paperpile.com/b/PyavUR/uzQSu)[10.1016/j.ecoinf.2020.101198](http://dx.doi.org/10.1016/j.ecoinf.2020.101198)

[Pouteau, R., Meyer, J.-Y., Taputuarai, R., & Stoll, B. (2012). Support vector machines to map rare and endangered native plants in Pacific islands forests. *Ecological Informatics*, *9*, 37–46. https://doi.org/](http://paperpile.com/b/PyavUR/sSBwV)[10.1016/j.ecoinf.2012.03.003](http://dx.doi.org/10.1016/j.ecoinf.2012.03.003)

[Prentice, I. C., Cramer, W., Harrison, S. P., Leemans, R., Monserud, R. A., & Solomon, A. M. (1992). Special Paper: A Global Biome Model Based on Plant Physiology and Dominance, Soil Properties and Climate. *Journal of Biogeography*, *19*(2), 117–134. https://doi.org/](http://paperpile.com/b/PyavUR/VJ9K)[10.2307/2845499](http://dx.doi.org/10.2307/2845499)

[Rummukainen, M. (2010). State‐of‐the‐art with regional climate models. *Wiley Interdisciplinary Reviews. Climate Change*, *1*(1), 82–96. https://doi.org/](http://paperpile.com/b/PyavUR/ghBxA)[10.1002/wcc.8](http://dx.doi.org/10.1002/wcc.8)

[Senay, S. D., Worner, S. P., & Ikeda, T. (2013). Novel three-step pseudo-absence selection technique for improved species distribution modelling. *PloS One*, *8*(8), e71218. https://doi.org/](http://paperpile.com/b/PyavUR/I72yk)[10.1371/journal.pone.0071218](http://dx.doi.org/10.1371/journal.pone.0071218)

[Synes, N. W., & Osborne, P. E. (2011). Choice of predictor variables as a source of uncertainty in continental-scale species distribution modelling under climate change. *Global Ecology and Biogeography: A Journal of Macroecology*, *20*(6), 904–914. https://doi.org/](http://paperpile.com/b/PyavUR/hmHCc)[10.1111/j.1466-8238.2010.00635.x](http://dx.doi.org/10.1111/j.1466-8238.2010.00635.x)

[Tessarolo, G., Rangel, T. F., Araújo, M. B., & Hortal, J. (2014). Uncertainty associated with survey design in Species Distribution Models. *Diversity & Distributions*, *20*(11), 1258–1269. https://doi.org/](http://paperpile.com/b/PyavUR/bt1ld)[10.1111/ddi.12236](http://dx.doi.org/10.1111/ddi.12236)

[Tytar, V., & Baidashnikov, O. (2021). Associations between habitat quality and body size in the carpathian-podolian land snail vestia turgida: Species distribution model selection and assessment of performance. *Zoodiversity*, *55*(1), 25–40.](http://paperpile.com/b/PyavUR/OQm6V) https://doi.org/10.15407/zoo2021.01.025

[VanDerWal, J., Shoo, L. P., Graham, C., & Williams, S. E. (2009). Selecting pseudo-absence data for presence-only distribution modeling: How far should you stray from what you know? *Ecological Modelling*, *220*](http://paperpile.com/b/PyavUR/NOt7X)[(4), 589–594. https://doi.org/](file:///G:\My%20Drive\MAIN\RESEARCH%20PROJECTS\MS%20IN%20PREP\2024_Lazagabaster.et.al_EcolEvolution_Hartebeest_SDMs\EE%20submission_reviewed\ms\(4),%20589–594.%20https:\doi.org\)[10.1016/j.ecolmodel.2008.11.010](http://dx.doi.org/10.1016/j.ecolmodel.2008.11.010)

[Varela, S., Rodríguez, J., & Lobo, J. M. (2009). Is current climatic equilibrium a guarantee for the transferability of distribution model predictions? A case study of the spotted hyena. *Journal of Biogeography*, *36*(9), 1645–1655. https://doi.org/](http://paperpile.com/b/PyavUR/hxL8z)[10.1111/j.1365-2699.2009.02125.x](http://dx.doi.org/10.1111/j.1365-2699.2009.02125.x)

[Velazco, S. J. E., Rose, M. B., de Andrade, A. F. A., Minoli, I., & Franklin, J. (2022). flexsdm : An r package for supporting a comprehensive and flexible species distribution modelling workflow. *Methods in Ecology and Evolution*, *13*](http://paperpile.com/b/PyavUR/CJfi)[(8), 1661–1669. https://doi.org/](file:///G:\My%20Drive\MAIN\RESEARCH%20PROJECTS\MS%20IN%20PREP\2024_Lazagabaster.et.al_EcolEvolution_Hartebeest_SDMs\EE%20submission_reviewed\ms\(8),%201661–1669.%20https:\doi.org\)[10.1111/2041-210x.13874](http://dx.doi.org/10.1111/2041-210x.13874)

[Wisz, M. S., & Guisan, A. (2009). Do pseudo-absence selection strategies influence species distribution models and their predictions? An information-theoretic approach based on simulated data. *BMC Ecology*, *9*, 8. https://doi.org/](http://paperpile.com/b/PyavUR/NKrhw)[10.1186/1472-6785-9-8](http://dx.doi.org/10.1186/1472-6785-9-8)

[Yen, J. D. L., Thomson, J. R., Vesk, P. A., & Mac Nally, R. (2011). To what are woodland birds responding? Inference on relative importance of in-site habitat variables using several ensemble habitat modelling techniques. *Ecography*, *34*(6), 946–954. https://doi.org/](http://paperpile.com/b/PyavUR/7R9oJ)[10.1111/j.1600-0587.2011.06651.x](http://dx.doi.org/10.1111/j.1600-0587.2011.06651.x)

[Zizka, A., Silvestro, D., Andermann, T., Azevedo, J., Duarte Ritter, C., Edler, D., Farooq, H., Herdean, A., Ariza, M., Scharn, R., Svantesson, S., Wengström, N., Zizka, V., & Antonelli, A. (2019). CoordinateCleaner : Standardized cleaning of occurrence records from biological collection databases.](http://paperpile.com/b/PyavUR/iWJV) [*Methods in Ecology and Evolution*](file:///G:\My%20Drive\MAIN\RESEARCH%20PROJECTS\MS%20IN%20PREP\2024_Lazagabaster.et.al_EcolEvolution_Hartebeest_SDMs\EE%20submission_reviewed\ms\Methods%20in%20Ecology%20and%20Evolution)[, *10*(5), 744–751. https://doi.org/](http://paperpile.com/b/PyavUR/iWJV)[10.1111/2041-210x.13152](http://dx.doi.org/10.1111/2041-210x.13152)
